# Hydration conditions as a critical factor in antibiotic-mediated bacterial competition outcomes

**DOI:** 10.1128/aem.02004-24

**Published:** 2024-12-23

**Authors:** Yana Beizman-Magen, Tomer Orevi, Nadav Kashtan

**Affiliations:** 1 Institute of Environmental Sciences, Department of Plant Pathology and Microbiology, Robert H. Smith Faculty of Agriculture, Food, and Environment, Hebrew University 54621 , Rehovot, Israel; University of Illinois Urbana-Champaign, Urbana, Illinois, USA

**Keywords:** bacterial interactions, interspecies competition, hydration conditions, microbial ecology, antibiotic-mediated competition, microscopic surface wetness, environmental microbiology, biological control

## Abstract

Antibiotic secretion plays a pivotal role in bacterial interference competition; yet, the impact of environmental hydration conditions on such competition is not well understood. Here, we investigate how hydration conditions affect interference competition among bacteria, studying the interactions between the antibiotic-producing *Bacillus velezensis* FZB42 and two bacterial strains susceptible to its antibiotics: *Xanthomonas euvesicatoria* 85-10 and *Pseudomonas syringae* DC3000. Our results show that wet-dry cycles significantly modify the response of the susceptible bacteria to both the supernatant and cells of the antibiotic-producing bacteria, compared to constantly wet conditions. Notably*, X. euvesicatoria* shows increased protection against both the cells and supernatants of *B. velezensis* under wet-dry cycles, while *P. syringae* cells become more susceptible under wet-dry cycles. In addition, we observed a reciprocal interaction between *P. syringae* and *B. velezensis*, where *P. syringae* inhibits *B. velezensis* under wet conditions. Our findings highlight the important role of hydration conditions in shaping bacterial interference competition, providing valuable insights into the microbial ecology of water-unsaturated surfaces, with implications for applications such as biological control of plant pathogens and mitigating antibiotic resistance.

**IMPORTANCE:**

Our study reveals that hydration conditions, particularly wet-dry cycles, significantly influence antibiotic-mediated competition between bacterial species. We revealed that the effectiveness of antibiotics produced by *Bacillus velezensis* against two susceptible bacterial species: *Xanthomonas* and *Pseudomonas* varies based on these hydration conditions. Unlike traditional laboratory environments, many real-world habitats, such as soil, plant surfaces, and even animal skin, undergo frequent wet-dry cycles. These conditions affect bacterial competition dynamics and outcomes, with wet-dry cycles providing increased protection for some bacteria while making others more susceptible. Our findings highlight the importance of considering environmental hydration when studying microbial interactions and developing biological control strategies. This research has important implications for improving agricultural practices and understanding natural microbial ecosystems.

## INTRODUCTION

Bacteria, ubiquitous members of every ecosystem on our planet, play important roles in the maintenance and functioning of ecological processes (1–3). These microorganisms do not live in isolation, but within complex communities, where interactions among different species are common (4, 5). The intricate interactions among bacterial species play a large role in shaping community composition and dynamics (4, 6–10). Such interactions underlie important features of microbial communities such as diversity and stability (9, 10). Deciphering the nature and outcomes of these interactions is crucial for understanding the complexities of microbial ecology and its impact on ecological systems and processes (4, 9).

Bacterial interspecies competition is a common interaction that can take on active or passive forms (11–13). The passive form involves competition for resources such as nutrients or space, whereas active competition involves one species secreting compounds that actively inhibit the growth or kill other species. This type of competition is called interference competition (11, 13, 14). While interference competition commonly occurs naturally, it can also be artificially induced, as in the application of bacterial biological control against plant pathogens (12, 15), or in bioremediation practices (16).

The outcome and dynamics of most interspecific interactions among bacteria can be influenced by the chemical, physical, and biological characteristics of the environment. Factors such as nutrient availability, temperature, pH, and surface type significantly affect these interactions (17–22). Among these factors, hydration conditions—which refer to the availability of water or moisture, ranging from extremely dry to fully saturated environments—play a pivotal role in many terrestrial microbial habitats. Hydration conditions are governed by the complex interplay of the presence of water or vapor, relative humidity, temperature, and the chemical and physical characteristics of surfaces (23–29). Typically, in environments that are not constantly saturated with water, hydration conditions are not static, but rather fluctuate dynamically, often characterized by recurring wet-dry cycles (30, 31).

During the “dry” phases of wet-dry cycles, surfaces can retain microscopic surface wetness (MSW [25, 30, 31]). MSW can take the form of microscopic droplets or thin liquid films, often invisible to the naked eye. A primary factor driving MSW formation and retention on surfaces is the presence of deliquescent substances, such as highly hygroscopic salts. For example, on leaf surfaces, a major source of salts comes from atmospheric aerosols present ubiquitously across plant foliage globally (25, 30). These salts can form, or retain, microscopic wetness when the relative humidity is above the deliquescence, or efflorescence, points (25, 30). MSW is prevalent on both biotic and abiotic surfaces (25, 31–34) and has been shown to often form around bacterial aggregates and other microorganisms (25, 31). Moreover, MSW has been found to protect bacteria and other microorganisms from desiccation and affect their survival rates (25, 31, 35). Microscopic wetness has been shown to significantly impact bacterial life and ecology, affecting mobility, communication, horizontal gene transfer (36), and spatial organization during colonization of surfaces (25, 37).

In a recent study, we have revealed that wet-dry cycles with MSW can protect bacteria from major antibiotic classes (38). We found that under wet-dry cycles, bacterial response to major antibiotic classes, markedly differs from their reaction under constantly wet conditions. Under wet-dry cycles with a period of MSW conditions (at the “dry” period), bacteria showed increased protection from diverse antibiotic classes, mostly antibiotic classes that are effective against actively growing cells. Through a combination of experiments and computational modeling, we suggested four mechanisms, operating at different phases of wet-dry cycles, that lead to increased protection from antibiotics under wet-dry cycles: (i) cross-protection due to high salt concentrations, (ii) “tolerance by slow growth,” (iii) deactivation of antibiotics by the physicochemical conditions associated with drying and MSW, and (iv) “tolerance by lag” during rewetting (38). Given that hydration conditions and the presence of MSW notably affect bacterial responses to antibiotics, we hypothesize that these factors may also exert an impact on antibiotic-mediated bacterial interference competition outcomes. Throughout this study, we adhere to the definition of antibiotics or antimicrobials as compounds produced by one microorganism that inhibit or kill another microorganism (39, 40).

To explore how wet-dry cycles with MSW affect interference competition among bacteria, we selected the biological control agent *Bacillus velezensis* FZB42 (*Bv*FZB42) (41), renowned for its ability to produce a broad spectrum of antimicrobial compounds (41) (Table S1). These antimicrobial compounds produced by *Bv*FZB42 include a variety of secondary metabolites that can affect bacterial and fungal pathogens. Key metabolites include lipopeptides (surfactin, bacillomycin D, and fengycin) and polyketides (bacillaene, difficidin, and macrolactin) (41–43). Surfactin plays a role in inducing systemic resistance in plants, while bacillomycin D and fengycin are highly effective against fungi, causing morphological changes and cell death (44, 45), and also exhibit activity against bacteria by cell membrane disruption (46). Polyketides like bacillaene and difficidin possess potent antibacterial properties (42, 44, 47). In addition, *Bv*FZB42 produces bacilysin, which has antimicrobial effects on both Gram-positive and Gram-negative bacteria (43).

As competition partners for *Bv*FZB42, we chose two well-studied plant foliar pathogens: *Xanthomonas euvesicatoria* pv. vesicatoria 85-10 *(Xee*85-10) (48, 49), and *Pseudomonas syringae* DC3000 (*Pst*DC3000) (50). Secondary metabolites produced by *BvFZB42* can affect these or similar species. Notably, three of these antibiotic compounds—difficidin, bacillaene, and bacilysin —are effective against *Xanthomonas* strains (47, 51, 52). Difficidin, a macrolide, inhibits protein synthesis by binding to ribosomal subunits (53), and may also compromise cell membranes (54). Similarly, bacillaene also inhibits protein synthesis through diverse mechanisms (55) and was found to play a major role in the inhibition of *Pseudomonas chlororaphis* by interacting with the protein elongation factor FusA (56). Bacilysin, on the other hand, disrupts peptidoglycan biosynthesis through anticapsin, impacting bacterial growth in a similar way to beta-lactam antibiotics (57, 58).

We designed an experimental setup that enables the incubation of bacterial cultures within droplets of various sizes, maintained either wet (at 100% relative humidity) or subjected to wet-dry cycles (at 85% relative humidity) featuring MSW periods. We hypothesize that the response of antibiotic-sensitive bacteria, to antibiotic-producing bacteria, under a wet-dry cycle with MSW, can be significantly different from their response under constantly wet conditions. We incubated cells of *Xee*85-10 or *Pst*DC3000 in droplets of various sizes, either alone or in the presence of *Bv*FZB42 supernatants, cells, or both. Experiments were conducted under wet-dry cycle conditions or constantly wet conditions. At the end of the experiment, cells were extracted from the drops and plated on agar plates and CFUs were counted to determine the competition outcomes.

## RESULTS

### Inhibition of *Xee*85-10 and *Pst*DC3000 by *Bv*FZB42 supernatants and cells under standard laboratory assays

**Fig 1:**
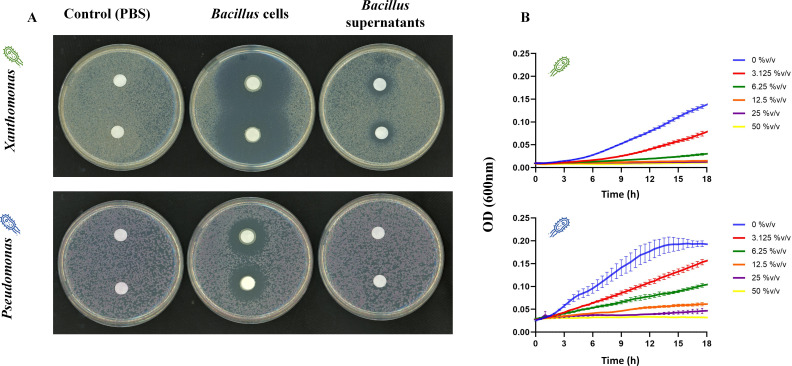
*Bacillus velezensis* FZB42 cells or supernatants inhibit *Xanthomonas euvesicatoria* 85-10 and *Pseudomonas syringae* DC3000. (**A**) Inhibition zone assay of *Xee*85-10 (top) and *Pst*DC3000 (bottom) by *Bv*FZB42 supernatants (pure 100% supernatants solution, right), cells (OD_600_ = 0.1, center) and control (PBS, left). Inhibition is more effective as the halo zone around the discs is larger. (**B**) Determination of MIC of *Xee*85-10 (top) and *Pst*DC3000 (bottom) by *Bv*FZB42 supernatants. Each line and error bars represent the mean ± SE of three repeats (see Materials and Methods).

Initially, we sought to determine the inhibitory capabilities of *Bv*FZB42 cells and/or their supernatants (see Materials and Methods) against *Xee*85-10 and *Pst*DC3000. Through two established assays—an inhibition zone assay and a MIC assay (specifically for supernatants)—we observed that both the cells and the supernatants of *Bv*FZB42 exerted a significant antagonistic effect against both *Xee*85-10 and *Pst*DC3000. This effect was evident in the results of inhibition zone assays (Fig. 1A), as well as MIC assays (as depicted in Fig. 1B).

Notably, *Pst*DC3000 was inhibited in 50% vol/vol concentration of *Bv*FZB42 supernatants (meaning when the volume consisted of 50% *Bv*FZB42 supernatants), while *Xee*85-10 required a lower concentration of 12.5% vol/vol for inhibition.

### Design of an experimental setup for bacterial culturing under constant wetness or wet-dry cycle conditions

To explore the effects of hydration conditions on bacterial interference competition, our experimental setup involved culturing bacteria within drying droplets, as detailed in Materials and Methods. A key variable influencing the drying dynamics of these droplets is their initial size. To comprehensively investigate a range of drying dynamics and corresponding “drying times,” we utilized droplets varying in volume from 1 µL to 100 µL. This range was selected to represent a spectrum of drying durations under controlled conditions (under moderate relative humidity). Our observations confirmed that droplet volume dictates the time required to reach MSW conditions, with a short drying duration of ~1 h in the smallest droplets and longer drying times of ~8 h in the largest droplets (Fig. S1).

To assess and compare the influence of continuous wet conditions versus a 24 h wet-dry cycle, we maintained a subset of droplets at a constant humidity of 100% RH, ensuring they remained static (i.e., minimizing droplets’ evaporation) throughout the experiment. In parallel, a second subset of droplets was subjected to a cycle of drying (under moderate relative humidity, RH = 85%) until stable MSW formed and followed by rewetting. This design allowed us to closely monitor and analyze the effects of hydration dynamics on the competition outcome (by CFU plating at the beginning and end of the experiments) under these distinct environmental conditions.

### *Xee*85-10 is less affected by *Bv*FZB42 supernatants under a wet-dry cycle compared to constantly wet conditions

**Fig 2:**
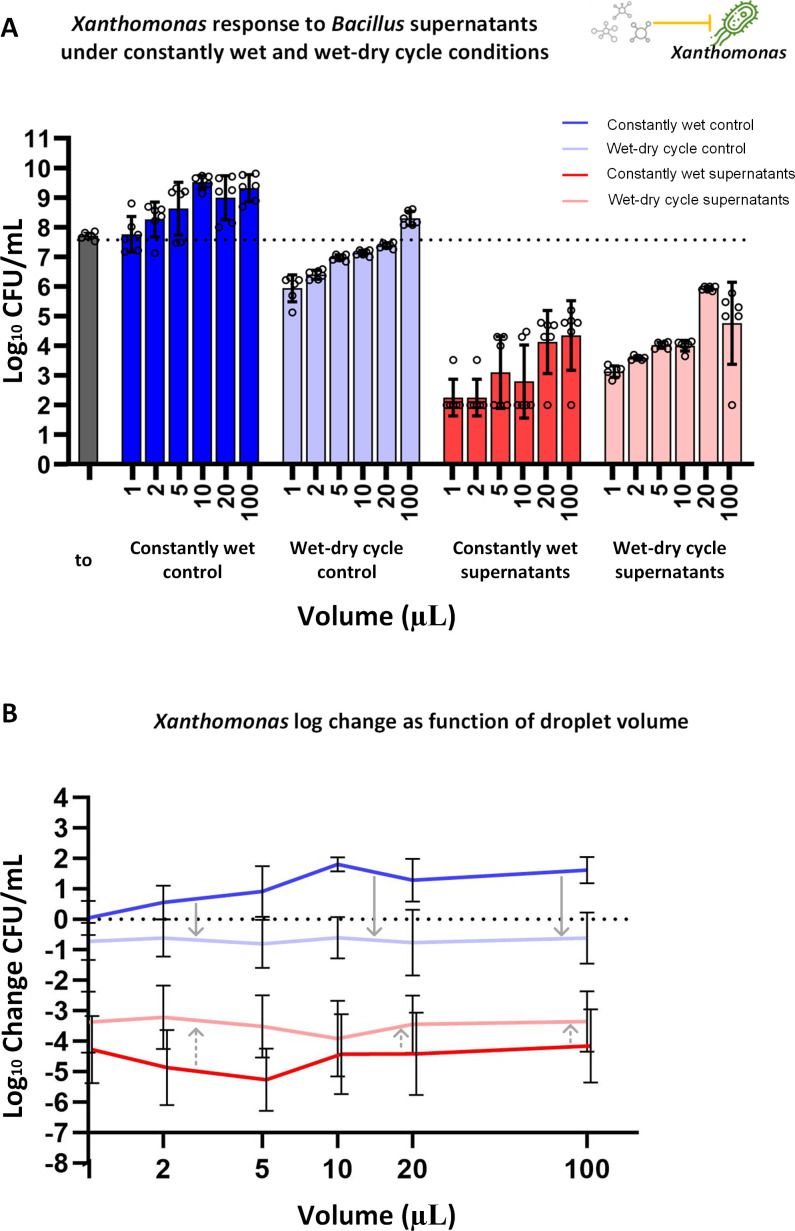
*Xanthomonas euvesicatoria* response to *Bacillus velezensis* supernatants under wet-dry cycle and constantly wet conditions. (**A**) Log_10_ CFU/mL of *Xee*85-10 exposed to *Bv*FZB42 supernatants under constantly wet and wet-dry cycle conditions, at t = 24 h, at five different droplet volumes. The most left gray bar represents log_10_ CFU/mL at t = 0 h. Bars and error bars represent mean  ±  SE log_10_ CFU/mL. Black circles represent technical replicates. CFU measurements were conducted as described in Materials and Methods. One-way ANOVA was performed to compare the means of all conditions with each other (Fig. S2). (**B**) Log change in CFU/mL (log_10_ CFU/mL at t = 24 h minus log_10_ CFU/mL at t = 0 h), as a function of droplet size (X-axis in log scale), in the presence and absence of supernatants. Lines and error bars represent mean ± SD. Linear regression and Spearman correlation were performed for statistical assessment (Fig. S3). Gray arrows represent the changes between the wet-dry cycle and constantly wet conditions in control (solid arrows) and with the addition of supernatants (dashed arrows).

First, we established a baseline by evaluating *Xee*85-10’s growth over 24 h without the presence of *Bv*FZB42’s supernatants. Under constantly wet conditions, *Xee*85-10 exhibited up to a 100-fold increase in CFU in the larger droplet volumes, indicating robust growth capacity in an environment free from desiccation stress (one-way ANOVA, *P* < 0.05, Fig. 2A; Fig. S2). By contrast, under a wet-dry cycle, a different pattern emerged. In all droplet volumes except for the largest (100 µL), we observed a reduction in *Xee*85-10 CFUs, which suggests that the bacterium’s survival was adversely affected by the cyclic drying and rewetting process. This impact was more pronounced in smaller droplets indicating that the faster drying dynamics of the smaller droplets reduced cell viability (one-way ANOVA, *P* < 0.05, Fig. 2; Fig. S2 and S3).

Upon the addition of *Bv*FZB42 supernatants at a concentration of 30% vol/vol (Materials and Methods), a notable reduction in CFU, of ~3–5 orders of magnitude, was observed under constantly wet conditions, aligning with expectations set by previous inhibition assays (Fig. 1) and given that the supernatant concentration exceeded the MIC of 12.5% vol/vol (one-way ANOVA, *P* < 0.0005, Fig. 2A; Fig. S2). Under the wet-dry cycle, cells in all drop volumes demonstrated a significant CFU reduction from the baseline (t = 0 h) (ANOVA, *P* < 0.05, Fig. 2A; Fig. S2).

Notably, the CFU reduction was less pronounced under wet-dry cycles when supernatants were introduced, compared to the scenario without supernatant (ANOVA, *P* < 0.05, Fig. 2A and B; Fig. S2). These results show that *Xee*85-10 is more protected from *Bv*FZB42 supernatants under wet-dry conditions relative to constant wetness.

### The effectiveness of *Bv*FZB42 supernatants against *Pst*DC3000 was markedly more pronounced under a wet-dry cycle as opposed to constantly wet conditions

**Fig 3:**
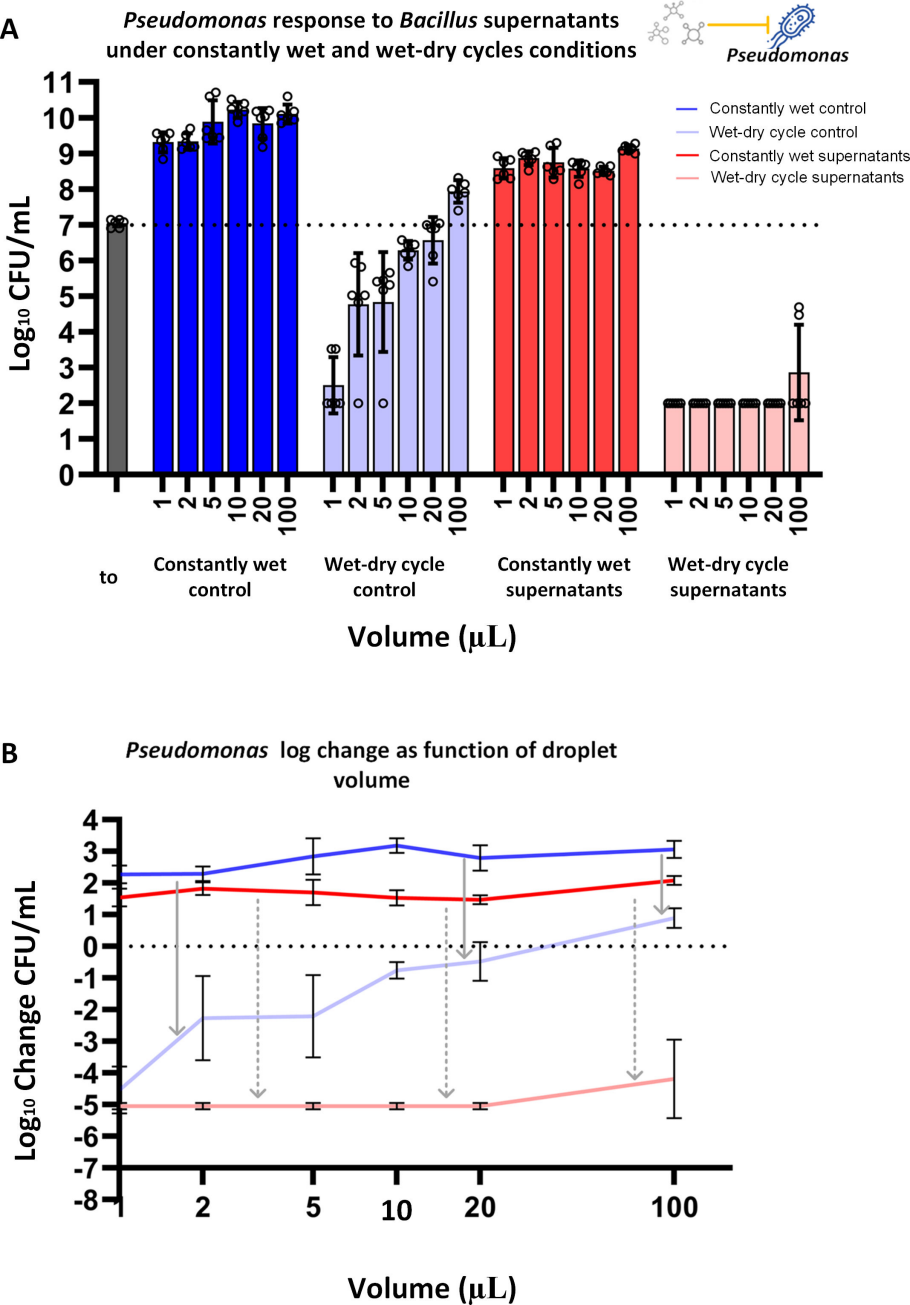
*Pseudomonas syringae* response to *Bacillus velezensis* supernatants under wet-dry cycle and constantly wet conditions. (**A**) Log_10_ CFU/mL of *Pst*DC3000 exposed to *Bv*FZB42 supernatants under constantly wet and wet-dry cycle conditions, at t = 24 h, at five different droplet volumes. The most left gray bar represents log_10_ CFU/mL at t = 0 h. Bars and error bars represent mean  ±  SE log_10_ CFU/mL. Black circles represent technical replicates. CFU measurements were conducted as described in Materials and Methods. One-way ANOVA was performed to compare the means of all conditions with each other (Fig. S4). (**B**) Log change in CFU/mL (log_10_ CFU/mL at t = 24 h minus log_10_ CFU/mL at t = 0 h), as a function of droplet size (X-axis in log scale), in the presence and absence of supernatants. Lines and error bars represent mean ± SD. Linear regression and Spearman correlation were performed for statistical assessment (Fig. S5). Gray arrows represent the changes between the wet-dry cycle and constantly wet conditions in control (solid arrows) and with the addition of supernatants (dashed arrows).

Without supernatants, *Pst*DC3000 CFU counts increased under constantly wet conditions and decreased under wet-dry cycles, with a larger decrease in smaller droplets (Fig. 3; Fig. S4 and S5), similar to the response pattern of *Xee*85-10. This pattern suggests a general decline in bacterial viability under the stress of wet-dry cycles, although larger droplets show an improved survival rate (one-way ANOVA, *P* < 0.05, Fig. 3; Fig. S4).

Introducing *Bv*FZB42 supernatants at a 30% vol/vol concentration (an equal concentration to the treatment for *Xee*85-10) elicited a distinct response in *Pst*DC3000. Under constant wetness, *Pst*DC3000’s CFU levels still increased from the baseline, as could be anticipated, given the supernatant concentration was below the MIC for *Pst*DC3000 (30% vol/vol versus a MIC of 50% vol/vol) (Fig. 3A; Fig. S4). Intriguingly, the wet-dry cycle conditions triggered a dramatic CFU reduction across all droplet volumes, much more than under constant wet conditions (one-way ANOVA, *P* < 0.0001, Fig. 3A; Fig. S4), as well as in comparison to wet-dry cycle without supernatants (one-way ANOVA, *P* < 0.0001, Fig. 3A; Fig. S4). Thus, *Pst*DC3000 cells were much more affected by *Bv*FZB42 supernatants under a wet-dry cycle compared to constantly wet conditions.

### Co-culture experiments representing two ecological scenarios

Next, we introduced an additional layer of complexity by performing co-culture experiments of the sensitive bacteria together with cells of the antibiotic-producing bacteria, *Bv*FZB42, with or without its supernatants. We employed two types of co-culture experiments; each represents a different ecological scenario. In both scenarios, there were similar concentrations of both *Bv*FZB42 and the sensitive bacteria at t = 0 h (~10^7^ CFU/mL). In the first, cells from *Bv*FZB42 and the pathogen (*Xee*85-10 or *Pst*DC3000) were co-cultured at an initial 1:1 cell ratio, simulating their concurrent arrival in a habitat (we term this scenario 1:1). The second scenario included both *Bv*FZB42 cells and its supernatants, resembling a situation where *Bv*FZB42 was already established and producing antibiotics before the pathogen’s arrival (we term this scenario 1:1 plus supernatants; 1:1 cell ratio of both species plus supernatants at t = 0). This setup also resembles biological control practices, such as foliar spraying, where both live microbial agents and their products are applied together (59–62).

### *Xee*85-10 is less affected by *Bv*FZB42 cells under a wet-dry cycle, with and without supernatants

**Fig 4:**
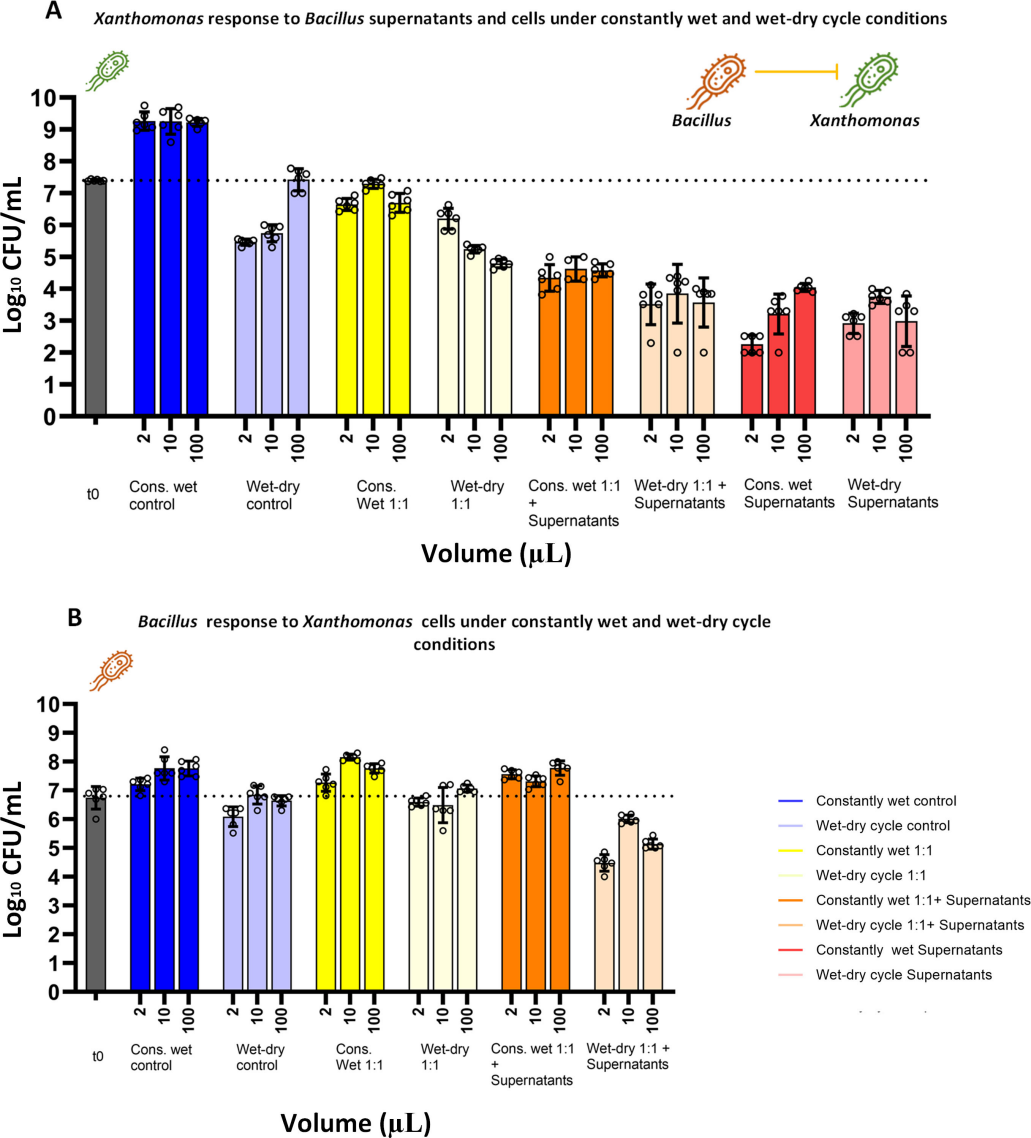
Co-culture experiments of *Xanthomonas euvesicatoria* and *Bacillus velezensis* under constantly wet and wet-dry cycle conditions. (**A**) Log_10_ CFU/mL of *Xee*85-10 at t = 24 h under both constantly wet and wet-dry cycle conditions across different co-culture scenarios: equal ratio of both bacteria (1:1); equal ratio of both bacteria with *Bv*FZB42 supernatants (1:1 + supernatants) and controls (*Xee*85-10 monoculture, *Bv*FZB42 monoculture, and *Xee*85-10 with *Bv*FZB42 supernatant) at three different droplet volumes. The left gray bar represents log_10_ CFU/mL at t = 0 h. Bars and error bars represent mean ± SE CFU/mL. Black circles represent technical replicates. One-way ANOVA was performed to compare the means between conditions and droplet volumes with each other (Fig. S6 and S8). (**B**) Same as in panel **A** but the bars represent *Bv*FZB42 CFU/mL (at t = 24 h).

Under constantly wet conditions, there was a decrease in *Xee*85-10 CFUs when *Bv*FZB42 cells were added with, or without, their supernatants (Fig. 4A). Co-culturing *Xee*85-10 with *Bv*FZB42 cells without supernatants showed a modest decrease in *Xee*85-10 CFUs under wet conditions, and a more significant reduction under wet-dry conditions. Adding supernatants to the mix increased the CFU reduction further, under both conditions. Interestingly, when comparing the effects of constant wetness to a wet-dry cycle with supernatants, CFU numbers were similar or slightly higher in the wet-dry cycle with supernatants only (Fig. 4A; Fig. S6). This observation reinforces the finding that *Xee*85-10 is less impacted by *Bv*FZB42 under wet-dry cycles.

The pronounced sensitivity of *Xee*85-10 to cells and supernatants of *Bv*FZB42 under constantly wet conditions is in agreement with suspended liquid co-cultures in microwells (quantified *via* plate reader, see Materials and Methods). In such microwell co-cultures, *Xee*85-10 growth was completely inhibited by *Bv*FZB42 cells, supernatants, or both (Fig. S7).

*Bv*FZB42, however, appeared to be unaffected by the presence of *Xee*85-10. Under both constantly wet and wet-dry cycle conditions, CFU counts were comparable in the monoculture and co-culture setups (control, 1:1, and 1:1 plus supernatants). Under constantly wet conditions, there was a significant increase in *Bv*FZB42 CFU counts (one-way ANOVA, *P* < 0.0001, Fig. 4B; Fig. S8). During a wet-dry cycle, a slight reduction in *Bv*FZB42 CFU counts was observed, which was more pronounced when supernatants were added (1:1 plus supernatants) (Fig. 4B). These results indicate that *Bv*FZB42’s growth, or survival, is not significantly affected by the presence of *Xee*85-10 in most conditions.

To enhance our understanding of the interactions between these two bacterial species, we have supplemented our previous data with area plots (Fig. S9). These plots offer a visual representation of changes in the abundance of each species within a competitive context. Under constantly wet conditions, *Xee*85-10 was generally outcompeted by *Bv*FZB42, particularly in the presence of supernatants with a clear decrease in *Xee*85-10’s abundance. During wet-dry cycles, the growth of both bacteria was inhibited regardless of supernatant presence (Fig. S9). In most scenarios, *Bv*FZB42 was the dominant competitor after 24 h. However, in the 1:1 scenario within small 2 µL droplets, *Xee*85-10 managed to outcompete *Bv*FZB42 (Fig. S9).

In summary, our findings suggest that *Bv*FZB42 inhibits *Xee*85-10 primarily due to the antibiotic compounds it secretes (present in its supernatants), yet this inhibitory effect is reduced under wet-dry cycles.

### The response of *Pst*DC3000 to *BvFZB42* cells and supernatants under the wet-dry cycle differed from that of *Xee*85-10

**Fig 5:**
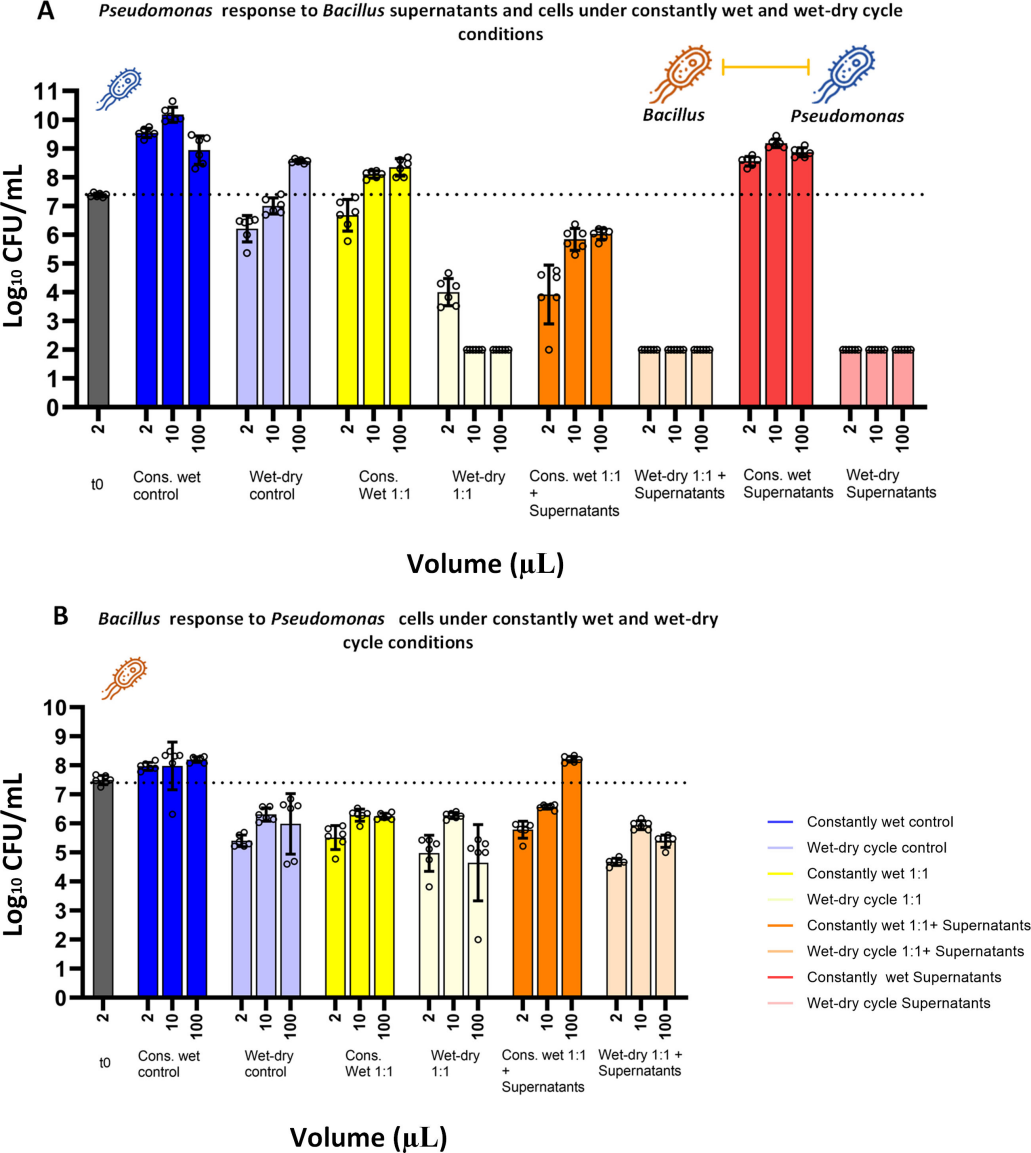
Co-culture experiments of *Pseudomonas syringae* and *Bacillus velezensis* under constantly wet and wet-dry cycle conditions. (**A**) Log_10_ CFU/mL of *Pst*DC3000 at t = 24 h under both constantly wet and wet-dry cycle conditions across different co-culture scenarios (equal ratio of both bacteria 1:1; equal ratio of both bacteria with *Bv*FZB42 supernatants 1:1 + supernatants) and controls (*Pst*DC3000 monoculture, *Bv*FZB42 monoculture, and *Pst*DC3000 with *Bv*FZB42 supernatant) at three different droplet volumes. The left gray bar represents log_10_ CFU/ml at t = 0 h. Bars and error bars represent mean ± SE CFU/mL. Black circles represent technical replicates. One-way ANOVA was performed to compare the means between conditions and droplet volumes with each other (Fig. S10 and S11). (**B**) Same as in panel **A** but the bars represent *Bv*FZB42 CFU/mL (at t = 24 h).

Under constantly wet conditions, *Pst*DC3000 exhibited a variable response when co-cultured with *Bv*FZB42. A moderate reduction in *Pst*DC3000 CFUs was observed in 1:1 co-culture with *Bv*FZB42 cells alone, which became more pronounced when both *Bv*FZB42 cells and their supernatant were present (Fig. 5A; Fig. S10). Remarkably, consistent with experiments using only supernatants (Fig. 3), under wet-dry cycles, there was a complete reduction of *Pst*DC3000 (resulting in zero observed CFU) in co-culture with *Bv*FZB42, regardless of the presence of supernatants. The response of *Bv*FZB42 to interaction with *Pst*DC3000 in co-culture differed significantly from that with *Xee*85-10. Under both constantly wet and wet-dry conditions, *Bv*FZB42 experienced significant inhibition, with the exception being under constantly wet conditions with supernatants in 10 and 100 µL droplets (one-way ANOVA, *P* < 0.005, Fig. 5B; Fig. S11).

These findings, revealing a complete reduction of *Pst*DC3000 under wet-dry cycles, were also very different from the results from co-cultures in suspended liquid cultures in microwells, where *Pst*DC3000 remained viable in the presence of both *Bv*FZB42 cells and supernatants (1:1 ratio) (Fig. S12), mirroring the outcomes observed under constantly wet conditions in droplet experiments. Under suspended liquid conditions, consistent with the droplet experiment, *Bv*FZB42 was inhibited. These observations further suggest that *Pst*DC3000 exerts an inhibitory effect on *Bv*FZB42, indicating a mutual inhibition between these two bacterial species (Fig. S12C and S13B).

The dynamics of competition between *Bv*FZB42 and *Pst*DC3000 in co-culture experiments reveal varied outcomes, as illustrated by the area plots (Fig. S14). Consistent with the above results, under constantly wet conditions in co-culture without supernatant, *Pst*DC3000 took over the population. This outcome was reversed in the large drops (100 µL) when supernatants were added, where *Bv*FZB42 outcompeted *Pst*DC3000. In scenarios involving wet-dry cycles, regardless of the supernatant presence, both *Pst*DC3000 and *Bv*FZB42 experienced a decline in CFUs. However, despite this reduction, the relative fraction of *Bv*FZB42 was larger (Fig. S14). These results highlight the significant impact of wet-dry cycles on the competition dynamics between these two bacterial species.

## DISCUSSION

In this study, we explored the interactions between the antibiotic-producing bacteria *Bv*FZB42 and two susceptible bacterial strains—*Xee*85-10 and *Pst*DC3000, under different hydration conditions, including constant wetness and wet-dry cycles. Our findings reveal that hydration conditions play a crucial role in determining the outcome of bacterial interference competition and the effectiveness of antibiotic-mediated interference. The differential responses of *Xee*85-10 and *Pst*DC3000 to *Bv*FZB42 cells and supernatants containing antibiotic compounds, highlight the potential for environmental factors, such as hydration conditions studied here, to modulate these interactions.

Our results demonstrate how hydration conditions critically influence the dynamics of antibiotic-mediated interference competition among bacteria, with the potential to either diminish or enhance the inhibitory effect. Notably, under constant wet conditions, *Xee*85-10 experienced inhibition by *Bv*FZB42, yet it showed increased protection from *Bv*FZB42’s supernatants during wet-dry cycles. By contrast, *Pst*DC3000 showed relative insensitivity to *Bv*FZB42’s supernatants under constantly wet environments but complete reduction under wet-dry cycles. This variation in competitive outcomes across different hydration scenarios highlights the importance of considering environmental conditions when predicting competition results, rather than relying only on standard laboratory assays in suspended liquid or on agar plates.

A deeper understanding of how hydration conditions modulate bacterial interactions can be achieved by characterizing the chemical components, such as antimicrobial molecules, involved in these interactions (47, 63–66). Further testing of the response of *Xee*85-10 and *Pst*DC3000 to the supernatants of two *Bv*FZB42 mutants that are deficient in the production of the antibiotics bacillaene and bacillomycin D demonstrated their involvement in these interspecies interactions. Specifically, we observed that supernatants of bacillaene-deficient mutant were less effective against both *Xee*85-10 and *Pst*DC3000 compared to the wild-type supernatants (at intermediate supernatant concentrations; Fig. S15). In addition, bacillomycin D-deficient mutants were less effective against *Pst*DC3000 than the wild-type supernatants (at the higher concentrations of supernatants, Fig. S15). However, at least one other antibiotic is likely involved in the interaction between *Bv*FZB42 and *Xee*85-10 or *Pst*DC3000, as the inhibition increased with higher supernatant concentrations. Comparable inhibition to the wild type was observed with both mutants’ supernatants at concentrations of 12.5% vol/vol and 75% vol/vol for *Xee*85-10 and *PstDC3000*, respectively (Fig. S15).

Thus, bacillaene is involved in the inhibition of both *Xee*85-10 and *Pst*DC3000, while bacillomycin D is involved solely in the inhibition of *Pst*DC3000. The participation of these two types of antibiotics in the studied interference interactions may explain the different effects that wet-dry cycles impose on the interactions between Bacillus and *Xee*85-10 or *Pst*DC3000 (Fig. 2 and 3). Bacillaene’s mode of action involves the inhibition of protein synthesis, and it is therefore mostly potent on metabolically active target cells (55, 67, 68). The increased protection of *Xee*85-10 from bacillaene and other antibiotic compounds produced by the Bacillus (Fig. 2) could stem from decreased susceptibility due to the slow or halted growth induced by drying, coupled with the low metabolic state associated with MSW conditions (38). This aligns with previous findings on the enhanced protection from beta-lactams under wet-dry cycles (38).

By contrast, both bacillaene and bacillomycin D produced by *Bv*FZB42 are involved in the inhibition of *Pst*DC3000. Given that bacillomycin D is a lipopeptide that damages bacterial membranes (44, 46), it is likely effective even against cells that are not actively growing, enabling it to kill cells during drying or under MSW conditions. In addition, another mechanism at play during drying is the dramatic increase of solute concentrations, including antibiotics, due to water evaporation. This process can potentially concentrate the active compounds beyond their MIC, thereby amplifying their lethal effects (25, 38). Bacillomycin D and possibly other antibiotic compound(s) from *Bv*FZB42, aside from bacillaene, that target *Pst*DC3000, might retain stability and activity, similar to certain antibiotics previously shown to maintain or even increase the effectiveness in such variable hydration environments (38). Thus, the specific mode of action of bacillomycin D, coupled with the elevated concentrations associated with drying, may explain the transition from insensitivity to eradication of *Pst*DC3000 under constant wetness and drying environments, respectively.

The involvement of antibiotics produced by *Bv*FZB42 in the inhibition of *Pst*DC3000 and *Xee*85-10 is further supported by the observation that commercially available antibiotics with similar modes of action to those produced by *Bv*FZB42 displayed comparable effects in wet-dry cycle experiments. Specifically, chloramphenicol (10 µg/mL) and norfloxacin (10 µg/mL) showed similar trends to *Bv*FZB42 supernatants in the case of *Xee*85-10 (protection under wet-dry cycles), while chloramphenicol (5 µg/mL) and erythromycin (5 µg/mL) had similar effects on *Pst*DC3000 (increased susceptibility under wet-dry cycle) (Fig. S16 and S17).

The mutual antagonism between *Bv*FZB42 and *Pst*DC3000, as observed in our study, has not been reported before to the best of our knowledge. Notably, our experiments demonstrated that *Pst*DC3000 is capable of inhibiting *Bv*FZB42 under both constant wetness and wet-dry cycles (Fig. 5; Fig. S13). The mechanism underlying the inhibition of *Bv*FZB42 by *Pst*DC3000 is unknown, though many works describe the antagonistic interactions between other *Bacillus* and *Pseudomonas* species (69, 70). These antagonistic interactions may include diverse mechanisms ranging from contact-dependent interactions such as T6SS to contact-independent interactions that are mediated by the production of different antimicrobial compounds (69, 70). One study also hints at a potential mechanism involving competitive behaviors linked to siderophore production (71).

Our study provides important insights into microbial ecology in water-unsaturated environments, but it has certain limitations. Our experiments, designed to simulate bacterial life on surfaces undergoing changes in wetness, were conducted using a medium that, while capturing some aspects of these conditions, lacks the complexity found on surfaces in natural microbial habitats (72, 73). Furthermore, while our findings suggest that mechanisms acting at the microscale, and populations with small census sizes within MSW droplets (74), may offer a potential explanation for some of the observed phenomena, we did not investigate these mechanisms at the single-cell, single-droplet level. Identifying and examining these microscale interactions represents an important direction for future research, which could provide valuable insights into the underlying processes.

This study highlights the profound influence of hydration conditions on interspecies bacterial interactions, in particular antibiotic-mediated interference competition. We observed that competition outcomes under wet-dry cycles can significantly differ from those in constantly wet environments. These variations are likely influenced by the unique properties of microscopic surface wetness—prevalent in terrestrial microbial habitats—and by its effect on bacterial physiology. Furthermore, the outcome of interactions in droplets may also diverge from those observed in standard laboratory experiments, whether in liquid suspension or on agar plates. Our research has potential implications for biocontrol applications of plant pathogens, suggesting that the dynamics observed could influence the effectiveness of biological control strategies. It shows that hydration conditions are important and that optimal hydration conditions can vary depending on the exact agent-pathogen pair. In conclusion, hydration conditions and their dynamics, in particular wet-dry cycles, play a pivotal role in shaping the outcomes of antibiotic-mediated competition among bacteria.

## MATERIALS AND METHODS

### Bacterial strains and growth conditions

The following bacterial strains were used in this study: *Xanthomonas euvesicatoria* pv. euvesicatoria 85-10, *Pseudomonas syringae* DC3000 (both strains obtained from the late Guido Sessa), both were transformed with the mCherry fluorescent plasmid pmp7605 (75) (transformation details below). *Bacillus velezenzis* FZB42_FB01 expressing gfp, *Bv*FZB42 ΔbmyA::EmR (defective in Bacillomycin D production), and BvFZB42 Δpks1KS1::cat (defective in bacillaene production) were obtained from Rainer Borriss, AmyloWiki (76).

At the beginning of each experiment, a small portion of the thawed glycerol stock was streaked on fresh LB agar + antibiotics plate (1 µg/mL Erytromycin for *B. velezensis*, 50 µg/mL Gentamicin sulfate for *Xee*85-10, and *Pst*DC3000). A single colony of each strain was inoculated into 5 mL of a fresh LB broth + appropriate antibiotic medium. Bacterial cultures were grown for 24 h under agitation set at 220 rpm, at 28°C. Then, each culture was centrifuged (2,935 rcf for 5 min) washed and resuspended in MTG medium (M9 × 1, glucose 4%, tryptone 1%) to reach an OD_600_ = 0.1 in 5 mL volume. Next, the cultures were grown for another 24 h under agitation set at 220  rpm, at 28°C.

The preparation of supernatants of *Bv*FB42 was done in a similar method to the one mentioned above. However, after centrifugation, the pellet was resuspended in 20 mL of MTG medium to reach OD_600_ = 0.1 and grown for 24 h in similar conditions. Then, the culture was centrifuged and filtered using a 0.22 µm filter to separate between the bacteria cells and their secreted products. Centrifugation and filtration were done twice. Supernatants were kept at 4°C for up to 4 days.

Each experiment was initiated with a bacterial inoculum of ≈ 5 × 10^7^ to 10^8^ CFU/mL.

### Electroporation

To transform both *Xee*85-10 and *Pst*DC3000 with pmp7605 plasmid (mCherry+), electrocompetent cells were prepared as described previously (77). The transformation was performed by electroporation (micropulser, BIO-RADUSA, program 1).

### Minimal inhibitory concentration and inhibition zone assays

To evaluate the antibacterial effect of *Bv*FZB42 cells and supernatants on *Xee*85-10 and *Pst*DC3000*,* MIC and inhibition zone assays were performed. To perform the MIC assay, cells were diluted into 96 microtiter plates (final OD_600_  =  0.06 for *Xee*85-10 and 0.2 for *Pst*DC3000) set with a twofold serial dilution of supernatants whereas the highest concentration of supernatants was 50% vol/vol. OD_600_ reads were taken every 30 min for 24 h using a plate reader (Synergy H1 Microplate Reader, BioTek Instruments, USA). MIC values were determined by the minimal concentration in which there was no significant bacterial growth. MIC assay was conducted before each experiment to validate that different supernatant batches had the same effect. The MIC chosen for all subsequent experiments, which will be described below, corresponds to 2.4 times the MIC determined for *Xee*85-10 (30% vol/vol).

For the inhibition zone assay, 1 mL of *Xee*85-10 and *Pst*DC3000 cells with an OD_600_ = 0.5 or 1, respectively, were serial diluted to $10^{-4}$, and evenly spread onto LB-agar plates (130 mm). After a 1 h incubation, discs containing deionized water (D_i_H_2_O), *Bv*FZB42 supernatants or live bacterial cells (OD_600_ = 0.5) were carefully placed on the inoculated plates. Subsequently, the plates were incubated at 28°C for 3 days, and the effectiveness of inhibition was qualitatively assessed by the presence of a clear area around the discs.

### Monoculture experiment with *Bv*FZB42 supernatants: wet-dry cycles and constantly wet conditions experimental setup

**Fig 6:**
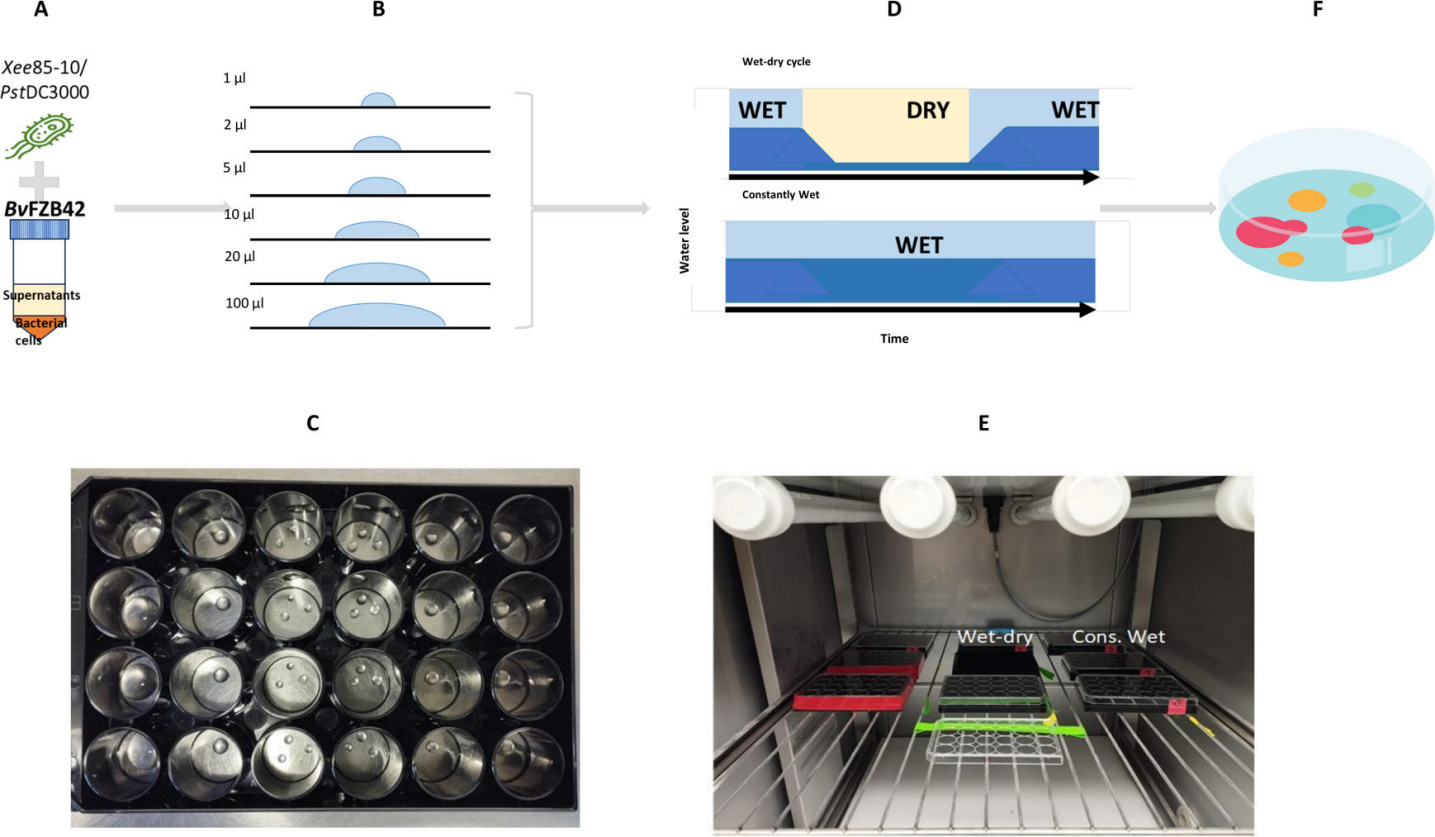
Experimental setup for droplet experiments under wet-dry cycle and constantly wet conditions. (**A**) In a monoculture experiment, one of the antibiotic-susceptible bacteria, either *Xee*85-10 or *Pst*DC3000 was mixed with 30% vol/vol of *Bv*FZB42 supernatants. (**B**) Then, droplets ranging from 1 to 100 µL volume were deposited into a 24-well plate (**C**). (**D**) Well plates were incubated in an environmental chamber with controlled temperature and relative humidity (**E**). (**F**) At the end of the experiment, droplets were re-suspended and plated on selective agar plates and CFU were counted.

Monoculture experiments were performed to evaluate the effect of a wet-dry cycle on *Xee*85-10 and *Pst*DC3000 response to the supernatants of *Bv*FZB42. Two conditions were investigated in these experiments, the first condition was bacteria without supernatants, as a control, and the second was bacteria (*Xee*85-10 or *Pst*DC3000) with 30% vol/vol supernatants (30% of the total volume was *Bv*FZB42 supernatants, prepared as described above). In these experiments, droplets of the following volumes: 1 µL, 2 µL, 5 µL, 10 µL, 20 µL, and 100 µL were deposited on the center of a well of a glass-bottom 24-well plate (24-well glass-bottom plate #1.5—Cellvis, USA). A total of six repeats (i.e., drops) was used for each tested drop volume. The 24-well plates were incubated in a growth chamber (Aralab, FITOCLIMA 600-PLH) for 24 h at 25°C and 85% relative humidity. To induce drying the wet-dry cycles plates were kept without their plastic lid. For constantly wet conditions, the empty cavities between the wells in these plates were filled with 300 µL of H_2_O, and the lid was sealed with tape, to maintain close to 100% relative humidity. The graphical representation of the monoculture experiment is presented in Fig. 6.

### Drying dynamics experiments

To characterize the drying dynamics of droplets of various volumes (1–100 µL), a microscopy method was employed by capturing time-lapse images of droplets’ area dynamics. The individual droplet was deposited onto the center of a well in a glass-bottom 24-well plate (#1.5 high-performance cover glass, Cellvis, USA). Three droplets (repeats) were used for each tested drop volume. The plate, without the lid, was then placed within a stage-top environmental control chamber (H301-K-FRAME, Okolab, Italy), pre-equilibrated to a temperature of 28°C, and a relative humidity of 75%. Images of the droplets were systematically captured every hour over a total duration of 22 h. The determination of droplet areas was performed using NIS elements software 5.03.

### Co-culture experiments: wet-dry cycle and constantly wet condition experimental setup

Co-culture experiments were performed to evaluate the effect of the wet-dry cycle on interference competition between *Bv*FZB42 and *Xee*85-10 or *Pst*DC3000. In this set of experiments, five conditions were investigated, two controls (monocultures of each strain of the pair), 1:1 ratio (*Xee*85-10 or *Pst*DC3000 cells in a similar concentration to *Bv*FZB42 cells), 1:1 ratio with 30% vol/vol supernatants and monoculture with 30% vol/vol supernatants. These experiments were conducted with three droplet volumes (2 µL, 10 µL, and 100 µL). Droplets were deposited on the center of a well of a glass-bottom 24-well. A total of six repeats (i.e., drops) were used for each volume. Incubation conditions for both wet-dry cycles and constantly wet treatments were identical to the monoculture experiments.

All five conditions were investigated, in parallel, also in well-mixed suspended liquid cultures. The experimental setting mirrored that of the MIC experiments, with similar treatments. For co-cultures, plate-reader experiments florescence was measured in addition to optical density (OD). For *Xee*85-10 and *Pst*DC3000, changes in fluorescence signal were measured (mCherry, Em579/Ex616). Similarly, for *Bv*FZB423 change in fluorescence signal was measured (GFP, Ex479/Em520).

### Gradual rewetting protocol

For the rewetting phase of the wet-dry cycle experiments, at time t = 24 h, the empty cavities between the wells were filled with 300 µL of H_2_O, and the lid was placed on the plate and sealed with tape. The sealed plate was incubated for 30 min at 28°C (leading to RH >95% inside the plate). By the end of this step, the “dried” droplets (in MSW form) adsorbed moisture from the humidified air by condensation and deliquescence. At this point, 30 µL of medium was added to each well, then the plates were sealed again and incubated for an additional 30 min. Finally, medium was added to get a dilution of $10^{-1}$, 30 µL was taken for serial dilutions and drop assay and the rest was plated on plates with LB agar with appropriate antibiotics. CFUs were counted 24 h or 48 h after plating for *Bv*FZB42 and *Pst*DC3000, respectively, because each requires a different amount of time to form visible colonies on agar plates.

Results from entire repetitions of these experiments are shown in Fig. S18 to 20.

### Statistical analysis

Comparisons of cell viability (based on CFU counts) among all conditions in wet-dry cycles and constantly wet conditions were conducted using one-way ANOVA, followed by Tukey’s test. The determination of correlations and relationships between droplet size and changes in cell viability (the difference between the number of CFUs at t = 24 h and the number of CFUs at t = 0 h) was performed using linear regression and Spearman correlation. Six technical replicates were performed for each condition in each experiment. All statistical analyses were performed using GraphPad Prism version 10.2.3 for Windows (GraphPad Software, USA).

## Data Availability

Data and code are available at 10.6084/m9.figshare.25998925. All data needed to evaluate the conclusions in the paper are present in the paper and/or the supplemental material.

## SUPPLEMENTAL MATERIAL

Supplemental Material aem.02004-24-s0001.pdf Table S1; Figures S1 to S20.

ASM does not own the copyrights to Supplemental Material that may be linked to, or accessed through, an article. The authors have granted ASM a non-exclusive, world-wide license to publish the Supplemental Material files. Please contact the corresponding author directly for reuse.
