# Supplementary material for: Hydration conditions as a critical factor in antibiotic-mediated bacterial competition outcomes
Source: Appl Environ Microbiol. 2024 Dec 23;91(1):e02004-24. doi: 10.1128/aem.02004-24 (PMC11784440; doi:10.1128/aem.02004-24)
Supplement: Supplemental Material — Table S1; Figures S1 to S20. [file aem.02004-24-s0001.pdf]

Supplemental Material

Hydration conditions as a critical factor in antibiotic-mediated bacterial competition outcomes

Yana Beizman-Magen[1], Tomer Orevi[1], Nadav Kashtan[1†]

SM Includes:

1. **Supplementary Table**
2. **Supplementary Figures (figures S1-S20)**
3. **References**

Antibacterial antibiotic produced by BvFZb42			Similar commercial antibiotic	
	**Name**	**Target**	**Name**	**Target**
**non-ribosomal lipopeptide**	Surfactin	Plasma membrane[1] , protein synthesis and enzymatic activity[2]	Daptomycin/ Polymyxin	Cell membrane[3,4]
	Bacillomycin D	Cell wall[5] and plasma membrane[6]	Daptomycin/ Polymyxin	Cell membrane[3,4]
	Fengycin	Plasma membrane[5] and cell integrity[7]	Daptomycin/ Polymyxin	Cell membrane[3,4]
	Iturin	Plasma membrane[8]	Daptomycin/ Polymyxin	Cell membrane[3,4]
	Bacillibactin	Targets iron uptake systems by binding ferric ions (Fe3+) and facilitating their transport into the cell[9]	Cefiderocol	Iron uptake[10]
	Bacilysin	cell wall (glucosamine-6-phosphate synthetase (G6PS))[9,11]	Fosfomycin/ Beta-lactams	Cell wall[12,13]
**non-ribosomal polyketides**	Macrolactin	Protein synthesis (peptide deformylase (PDF)) [14]	Chloramphenicol/ Erythromycin/ Tetracyclin/ Neomycin/	Protein synthesis[15–17]
	Bacillaene	Biofilm formation [18] and protein synthesis [19,20]	Norfloxacin /Chloramphenicol/ Erythromycin/ Tetracyclin/ Neomycin/	Biofilm formation, protein synthesis [15–17,21]
	Difficidin	DNA replication, protein production, and cell wall synthesis [22]	Norfloxacin/ Chloramphenicol/ Erythromycin/ Tetracyclin/ Neomycin/	Protein synthesis, DNA replication [15–17,21]

Table. S1. Literature based summary of identified antibiotics produced by BvFZB42 and their commercially available equivalent that work with similar modes of action [23].

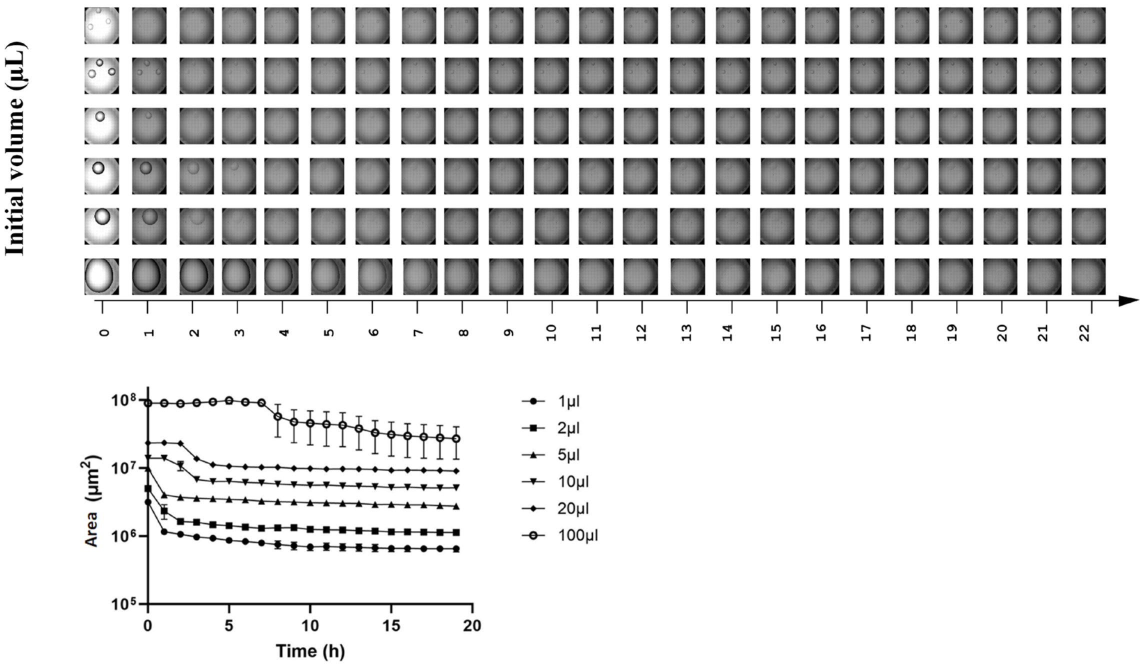

Fig. S1. Drying dynamics of MTG medium droplets of various volumes (1 µl to 100 µl).

(A) Droplets were incubated at 28°C and a relative humidity of 75%. Droplets were imaged at one hour interval over 22 hours. The cover surface area of the drop was used to track and estimate the drying dynamics. Each data point and error bar represent the mean ± SE area (μm^2) at a specific time points. The experiment details are described in the 'Methods' section. **(B)** Time-lapse micrographs of the drying process of droplets with various volumes (ranging 1-100 µL) over a period of 22 hours.

			Constantly wet control						Wet-dry cycle control						Constantly wet Spn						Wet-dry cycle Spn					
		t0	1	2	5	10	20	100	1	2	5	10	20	100	1	2	5	10	20	100	1	2	5	10	20	100
Constantly wet control	1	ns																								
	2	ns	ns																							
	5	ns	ns	ns																						
	10	****	**	ns	ns																					
	20	*	ns	ns	ns	ns																				
	100	***	*	ns	ns	ns	ns																			
Wet-dry cycle control	1	*	**	****	****	****	****	****																		
	2	ns	ns	***	****	****	****	****	ns																	
	5	ns	ns	ns	**	****	***	****	ns	ns																
	10	ns	ns	ns	*	****	***	****	ns	ns	ns															
	20	ns	ns	ns	ns	****	*	***	*	ns	ns	ns														
	100	ns	ns	ns	ns	ns	ns	ns	****	***	ns	ns	ns													
Constantly wet Spn	1	****	****	****	****	****	****	****	****	****	****	****	****	****												
	2	****	****	****	****	****	****	****	****	****	****	****	****	****	ns											
	5	****	****	****	****	****	****	****	****	****	****	****	****	****	ns	ns										
	10	****	****	****	****	****	****	****	****	****	****	****	****	****	ns	ns	ns									
	20	****	****	****	****	****	****	****	**	****	****	****	****	****	***	***	ns	ns								
	100	****	****	****	****	****	****	****	*	****	****	****	****	****	****	****	ns	*	ns							
Wet-dry cycle Spn	1	****	****	****	****	****	****	****	****	****	****	****	****	****	ns	ns	ns	ns	ns	ns						
	2	****	****	****	****	****	****	****	****	****	****	****	****	****	ns	ns	ns	ns	ns	ns	ns					
	5	****	****	****	****	****	****	****	***	****	****	****	****	****	**	**	ns	ns	ns	ns	ns	ns				
	10	****	****	****	****	****	****	****	***	****	****	****	****	****	**	**	ns	ns	ns	ns	ns	ns	ns			
	20	*	**	****	****	****	****	****	ns	ns	ns	ns	*	****	****	****	****	****	**	*	****	****	***	***		
	100	****	****	****	****	****	****	****	ns	**	****	****	****	****	****	****	**	***	ns	ns	**	ns	ns	ns	ns	

Fig. S2. Pairwise comparison of the mean CFU/mL (Log_{10} transformed) for each volume and treatment- *Xanthomonas* monoculture.

The significance was assessed by one-way ANOVA. Significance marked by *,**, *** or ****, denoting p-values of <0.05, <0.005, <0.0005 or <0.0001, respectively. Data points are same as presented in Fig. 2A. Analysis was performed using GraphPad Prism 10.

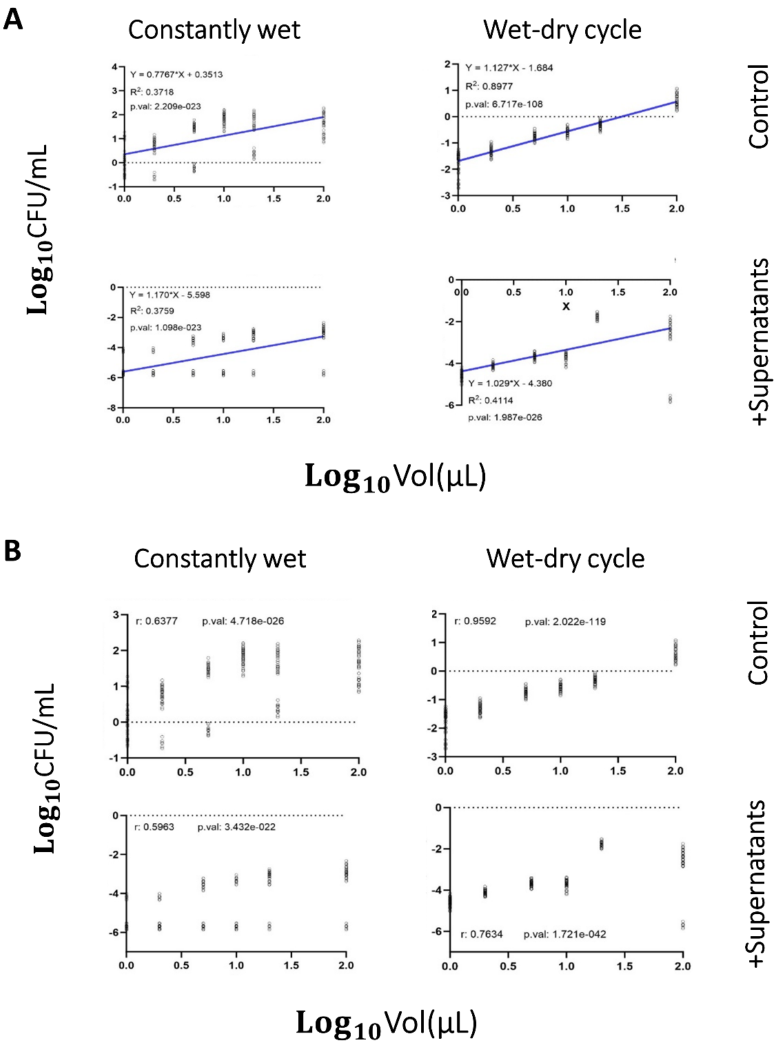

Fig. S3. Linear regression and spearman correlation of the change in *Xee*85-10 CFU/mL as function of volume, after 24 hours exposure to *Bv*FZB42 supernatants.

(**A**) linear regression analysis of the change of CFU/mL of *Xee*85-10 cells after exposure for 24 hours to supernatants produced by *Bv*FZB42 in constantly wet and wet-dry cycle conditions. Circles mark experimental values, regression line (blue line) is shown along with its linear equation, R^2 values and P-values. (**B**) Correlation between change in bacterial CFU/mL (Log_{10} transformed) and droplet volume (Log_{10} transformed). Circles mark experimental values. r values are Pearson correlation coefficients. Data points are same as presented in Fig. 2B. Analysis was performed in GraphPad Prism 10.

			Constantly wet control						Wet-dry cycle control						Constantly wet Spn						Wet-dry cycle Spn					
		t0	1	2	5	10	20	100	1	2	5	10	20	100	1	2	5	10	20	100	1	2	5	10	20	100
Constantly wet control	1	****																								
	2	****	ns																							
	5	****	ns	ns																						
	10	****	ns	ns	ns																					
	20	****	ns	ns	ns	ns																				
	100	****	ns	ns	ns	ns	ns																			
Wet-dry cycle control	1	****	****	****	****	****	****	****																		
	2	****	****	****	****	****	****	****	****																	
	5	****	****	****	****	****	****	****	****	ns																
	10	ns	****	****	****	****	****	****	****	**	**															
	20	ns	****	****	****	****	****	****	****	****	***	ns														
	100	ns	*	**	****	****	****	****	****	****	****	***	*													
Constantly wet Spn	1	**	ns	ns	*	***	*	**	****	****	****	****	****	ns												
	2	****	ns	ns	ns	*	ns	*	****	****	****	****	****	ns	ns											
	5	***	ns	ns	ns	**	ns	*	****	****	****	****	****	ns	ns	ns										
	10	**	ns	ns	*	***	*	**	****	****	****	****	****	ns	ns	ns	ns									
	20	**	ns	ns	*	***	*	**	****	****	****	****	****	ns	ns	ns	ns	ns								
	100	****	ns	ns	ns	ns	ns	ns	****	****	****	****	****	ns	ns	ns	ns	ns	ns							
Wet-dry cycle Spn	1	****	****	****	****	****	****	****	ns	****	****	****	****	****	****	****	****	****	****	****						
	2	****	****	****	****	****	****	****	ns	****	****	****	****	****	****	****	****	****	****	****	ns					
	5	****	****	****	****	****	****	****	ns	****	****	****	****	****	****	****	****	****	****	****	ns	ns				
	10	****	****	****	****	****	****	****	ns	****	****	****	****	****	****	****	****	****	****	****	ns	ns	ns			
	20	****	****	****	****	****	****	****	ns	****	****	****	****	****	****	****	****	****	****	****	ns	ns	ns	ns		
	100	****	****	****	****	****	****	****	ns	****	****	****	****	****	****	****	****	****	****	****	ns	ns	ns	ns	ns	

Fig. S4. Pairwise comparison of the mean CFU/mL (Log$_{10}$ transformed) for each volume and treatment - *Pseudomonas* monoculture.

The significance was assessed by one-way ANOVA. Significance marked *,**, *** or ****, denoting p-values of <0.05, <0.005, <0.0005 or <0.0001 respectively. Data points are same as presented in Fig. 3A. Analysis was performed in GraphPad Prism 10**.**

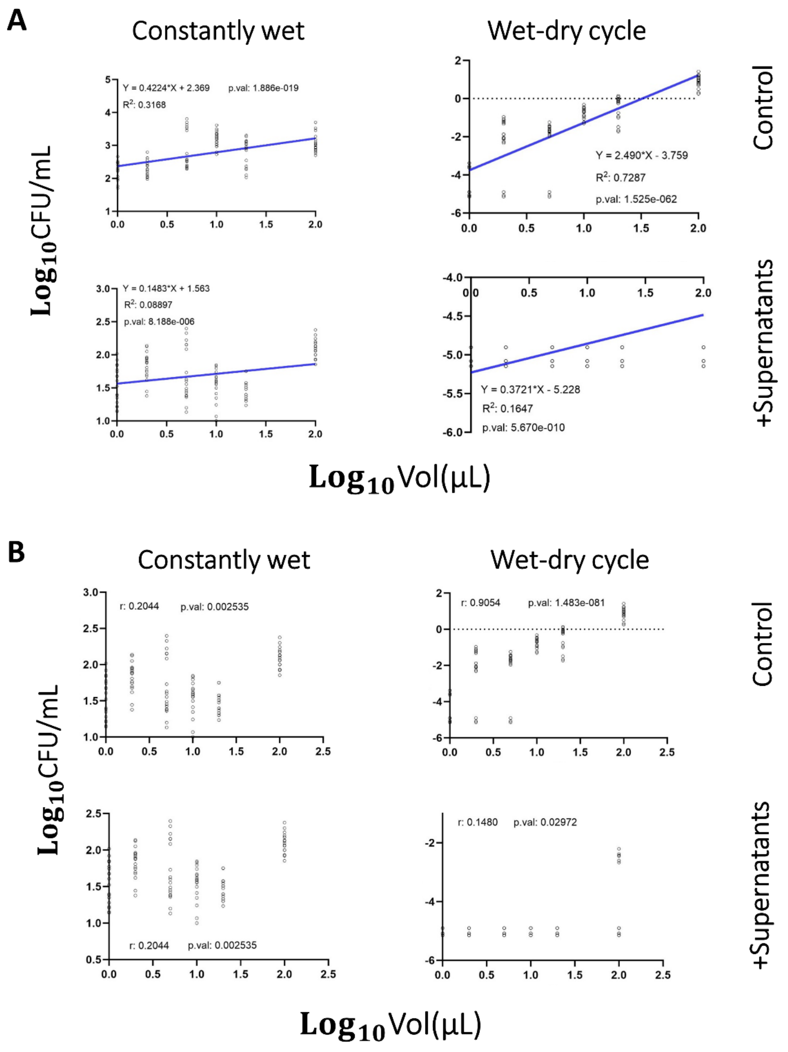

Fig. S5. Linear regression and spearman correlation of the change in *Pst*DC3000 CFU/mL as function of volume, after 24 hours exposure to *Bv*FZB42 supernatants.

(A) linear regression analysis of the change of CFU/mL of *Pst*DC3000 cells after exposure for 24 hours to supernatants produced by *Bv*FZB42 in constantly wet and wet-dry cycle conditions. Circles mark experimental values, regression line (blue line) is shown along with its linear equation, R^2 values and P-values. **(B)** Correlation between change in bacterial CFU/mL (Log_{10} transformed) and droplet volume (Log_{10} transformed). Circles mark experimental values. r values are Pearson correlation coefficients. Data points are same as presented in Fig. 3B. Analysis was performed in GraphPad Prism 10.

			constantly wet control			wet-dry cycle control			constntly wet 1:1			wet-dry cycle 1:1			constantly wet 1:1+Spn			wet-dry cycle 1:1+Spn			constantly wet Spn			wet-dry cycle Spn		
		t0	2	10	100	2	10	100	2	10	100	2	10	100	2	10	100	2	10	100	2	10	100	2	10	100
constant ly wet control	2	****																								
	10	****	ns																							
	100	****	ns	ns																						
wet-dry cycle control	2	****	****	****	****																					
	10	****	****	****	****	ns																				
	100	ns	****	****	****	****	****																			
constntl y wet 1:1	2	ns	****	****	****	*	ns	ns																		
	10	ns	****	****	****	****	****	ns	ns																	
	100	ns	****	****	****	**	ns	ns	ns	ns																
wet-dry cycle 1:1	2	**	****	****	****	ns	ns	**	ns	*	ns															
	10	****	****	****	****	ns	ns	****	***	****	***	ns														
	100	****	****	****	****	ns	ns	****	****	****	****	***	ns													
constant ly wet 1:1+Spn	2	****	****	****	****	*	***	****	****	****	****	****	ns	ns												
	10	****	****	****	****	****	****	****	****	****	****	****	***	ns	ns											
	100	****	****	****	****	ns	*	****	****	****	****	****	ns	ns	ns	ns										
wet-dry cycle 1:1+Spn	2	****	****	****	****	****	****	****	****	****	****	****	****	**	ns	ns	*									
	10	****	****	****	****	****	****	****	****	****	****	****	***	ns	ns	ns	ns	ns								
	100	****	****	****	****	****	****	****	****	****	****	****	****	**	ns	ns	ns	ns	ns							
constant ly wet Spn	2	****	****	****	****	****	****	****	****	****	****	****	****	****	****	***	****	**	****	**						
	10	****	****	****	****	****	****	****	****	****	****	****	****	****	*	ns	***	ns	ns	ns	ns					
	100	****	****	****	****	***	****	****	****	****	****	****	**	ns	ns	ns	ns	ns	ns	ns	****	ns				
wet-dry cycle Spn	2	****	****	****	****	****	****	****	****	****	****	****	****	****	***	ns	****	ns	ns	ns	ns	ns	*			
	10	****	****	****	****	****	****	****	****	****	****	****	***	ns	ns	ns	ns	ns	ns	ns	***	ns	ns	ns		
	100	****	****	****	****	****	****	****	****	****	****	****	****	****	**	ns	****	ns	ns	ns	ns	ns	*	ns	ns	

Fig. S6. Pairwise comparison of the mean CFU/mL (Log_{10} transformed) for each volume and treatment of *Xee*85-10 in co-culture with *Bv*FZB42.

The significance was assessed by one-way ANOVA. Significance marked by *,**, *** or ****, denoting p-values of <0.05, <0.005, <0.0005 or <0.0001, respectively. Data points are same as presented in Fig. 4A. Analysis was performed in GraphPad Prism 10.

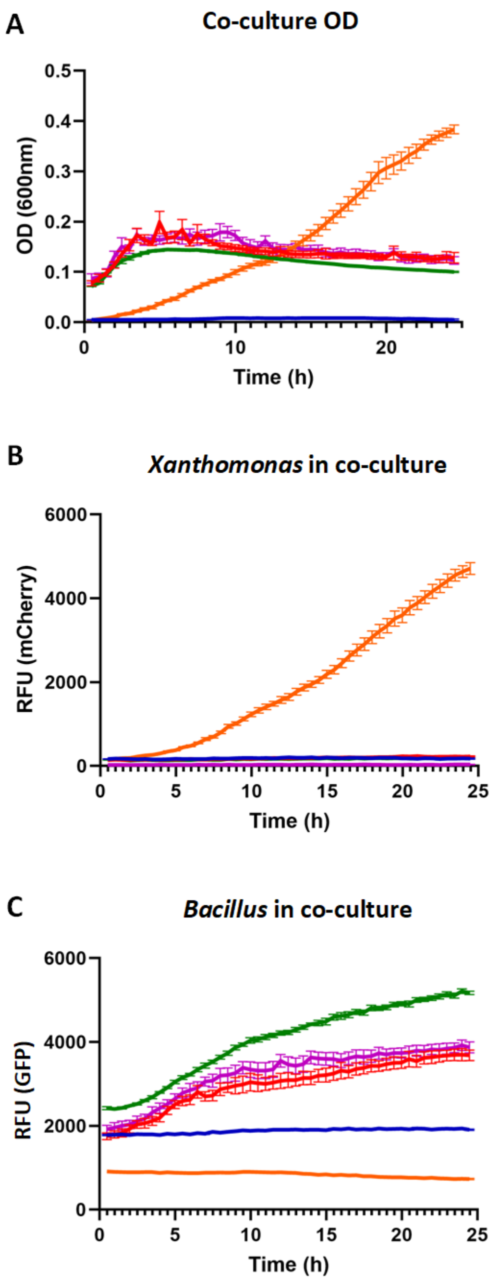

Fig. S7. Co-culture experiment of *Xee*85-10 and *Bv*FZB42 under continuous shaking.

A, B and C present the growth curves of the five different treatments in liquid bulk conditions. Four repeats of 200 µL of each treatment were grown in a continuous shaking environment at 28°C in the plate reader (Synergy H1 Microplate Reader, BioTek Instruments, USA). Measurements were taken every 30 minutes over a 24 hours duration. (A) change in optical density (both bacteria) (B) change in mCherry fluorescence signal (Growth of *Xee*85-10 in co-culture) (C) change in GFP fluorescence signal (growth of *Bv*FZB42 in co-culture). Lines and error bars represent mean ± SE.

			constantly wet control			wet-dry cycle control			constntly wet 1:1			wet-dry cycle 1:1			constantly wet 1:1+Spn			wet-dry cycle 1	
		t0	2	10	100	2	10	100	2	10	100	2	10	100	2	10	100	2	1
constantly wet control	2	ns																	
	10	****	ns																
	100	****	ns	ns															
wet-dry cycle control	2	*	****	****	****														
	10	ns	ns	****	****	**													
	100	ns	ns	****	****	ns	ns												
constntly wet 1:1	2	ns	ns	ns	ns	****	ns	*											
	10	****	****	ns	ns	****	****	****	****										
	100	****	ns	ns	ns	****	****	****	ns	ns									
wet-dry cycle 1:1	2	ns	*	****	****	ns	ns	ns	**	****	****								
	10	ns	**	****	****	ns	ns	ns	***	****	****	ns							
	100	ns	ns	**	**	****	ns	ns	ns	****	**	ns	ns						
constantly wet 1:1+Spn	2	***	ns	ns	ns	****	**	****	ns	*	ns	****	****	ns					
	10	ns	ns	ns	ns	****	ns	**	ns	***	ns	**	***	ns	ns				
	100	****	ns	ns	ns	****	****	****	ns	ns	ns	****	****	**	ns	ns			
wet-dry cycle 1:1+Spn	2	****	****	****	****	****	****	****	****	****	****	****	****	****	****	****	****		
	10	**	****	****	****	ns	***	*	****	****	****	*	ns	****	****	****	****	****	
	100	****	****	****	****	****	****	****	****	****	****	****	****	****	****	****	****	*	****

Fig. S8. Pairwise comparison of the mean CFU/mL (Log_{10} transformed) for each volume and treatment of *Bv*FZB42 in co-culture with *Xee*85-10.

The significance was assessed by one-way ANOVA. Significance marked by *,**, *** or ****, denoting p-values of <0.05, <0.005, <0.0005 or <0.0001, respectively. Data points are same as presented in Fig. 4B. Analysis was performed in GraphPad Prism 10.

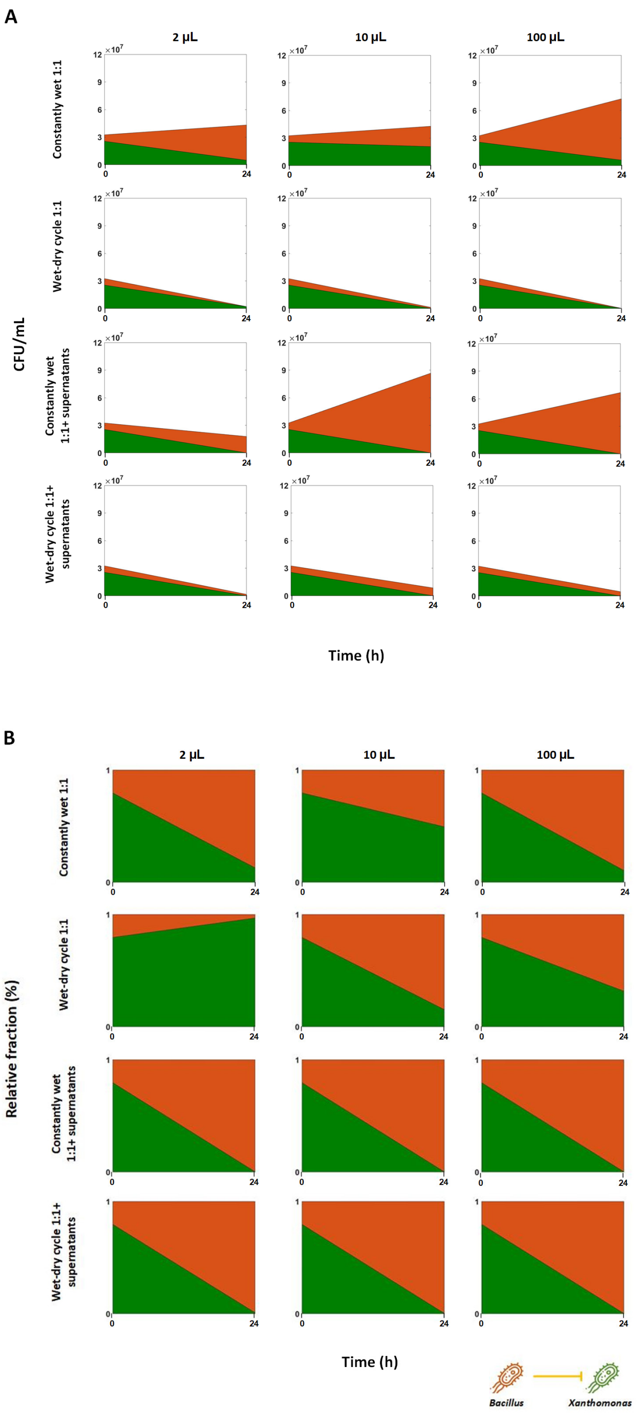

Fig. S9. Competition dynamics of *Xee*85-10 and *Bv*FZB42 co-culture in droplets.

(A) stacked area plots and (B) relative part area plots in droplet co-cultures (1:1 and 1:1+ supernatants). The green fraction represents *Xee*85-10, and the orange fraction represents *Bv*FZB42. Note the plot is based on only two time points (at t=0 h and t=24 h).

			constantly wet control			wet-dry cycle control			constntly wet 1:1			wet-dry cycle 1:1			constantly wet 1:1+Spn			wet-dry cycle 1:1+Spn			constantly wet Spn			wet-dry cycle Spn		
		t0	2	10	100	2	10	100	2	10	100	2	10	100	2	10	100	2	10	100	2	10	100	2	10	100
constantly wet control	2	****																								
	10	****	ns																							
	100	****	ns	****																						
wet-dry cycle control	2	****	****	****	****																					
	10	ns	****	****	****	**																				
	100	****	***	****	ns	****	****																			
constntly wet 1:1	2	*	****	****	****	ns	ns	****																		
	10	*	****	****	**	****	****	ns	****																	
	100	***	****	****	ns	****	****	ns	****	ns																
wet-dry cycle 1:1	2	****	****	****	****	****	****	****	****	****	****															
	10	****	****	****	****	****	****	****	****	****	****	****														
	100	****	****	****	****	****	****	****	****	****	****	****	ns													
constantly wet 1:1+Spn	2	****	****	****	****	****	****	****	****	****	****	ns	****	****												
	10	****	****	****	****	ns	****	****	**	****	****	****	****	****	****											
	100	****	****	****	****	ns	***	****	ns	****	****	****	****	****	****	ns										
wet-dry cycle 1:1+Spn	2	****	****	****	****	****	****	****	****	****	****	****	ns	ns	****	****	****									
	10	****	****	****	****	****	****	****	****	****	****	****	ns	ns	****	****	****	ns								
	100	****	****	****	****	****	****	****	****	****	****	****	ns	ns	****	****	****	ns	ns							
constantly wet Spn	2	****	***	****	ns	****	****	ns	****	ns	ns	****	****	****	****	****	****	****	****	****						
	10	****	ns	****	ns	****	****	ns	****	****	**	****	****	****	****	****	****	****	****	****	ns					
	100	****	ns	****	ns	****	****	ns	****	*	ns	****	****	****	****	****	****	****	****	****	ns	ns				
wet-dry cycle Spn	2	****	****	****	****	****	****	****	****	****	****	****	ns	ns	****	****	****	ns	ns	ns	****	****	****			
	10	****	****	****	****	****	****	****	****	****	****	****	ns	ns	****	****	****	ns	ns	ns	****	****	****	ns		
	100	****	****	****	****	****	****	****	****	****	****	****	ns	ns	****	****	****	ns	ns	ns	****	****	****	ns	ns	

Fig. S10. Pairwise comparison of the mean CFU/mL (Log_{10} transformed) for each volume and treatment of *Pst*DC3000 in co-culture with *Bv*FZB42.

The significance was assessed by one-way ANOVA. Significance marked by *,**, *** or ****, denoting p-values of <0.05, <0.005, <0.0005 or <0.0001, respectively. Data points are same as presented in Fig. 6A. Analysis was performed in GraphPad Prism 10.

		t0	constantly wet control			wet-dry cycle control			constntly wet 1:1			wet-dry cycle 1:1			constantly wet 1:1+Spn			wet-dry cycle 1:1+Spn		
			2	10	100	2	10	100	2	10	100	2	10	100	2	10	100	2	10	100
constantly wet control	2	ns																		
	10	ns	ns																	
	100	ns	ns	ns																
wet-dry cycle control	2	****	****	****	****															
	10	**	****	****	****	ns														
	100	****	****	****	****	ns	ns													
constntly wet 1:1	2	****	****	****	****	ns	ns	ns												
	10	**	****	****	****	ns	ns	ns	ns											
	100	**	****	****	****	ns	ns	ns	ns	ns										
wet-dry cycle 1:1	2	****	****	****	****	ns	***	*	ns	**	**									
	10	**	****	****	****	ns	ns	ns	ns	ns	ns	**								
	100	****	****	****	****	ns	****	***	ns	****	****	ns	****							
constantly wet 1:1+Spn	2	****	****	****	****	ns	ns	ns	ns	ns	ns	ns	ns	*						
	10	ns	***	***	****	**	ns	ns	*	ns	ns	****	ns	****	ns					
	100	ns	ns	ns	ns	****	****	****	****	****	****	****	****	****	****	****				
wet-dry cycle 1:1+Spn	2	****	****	****	****	ns	****	**	ns	****	****	ns	****	ns	*	****	****			
	10	****	****	****	****	ns	ns	ns	ns	ns	ns	ns	ns	**	ns	ns	****	**		
	100	****	****	****	****	ns	ns	ns	ns	ns	ns	ns	ns	ns	ns	**	****	ns	ns	

Fig. S11. Pairwise comparison of the mean CFU/mL (Log_{10} transformed) for each volume and treatment of *Bv*FZB42 in co-culture with *Pst*DC3000.

Pairwise comparison of the mean CFU/ml (Log_{10} transformed) of each volume in each treatment. The significance was assessed by one-way ANOVA. Significance marked by *,**, *** or ****, denoting p-values of <0.05, <0.005, <0.0005 or <0.0001, respectively. Data points are same as presented in Fig. 6B. Analysis was performed in GraphPad Prism 10.

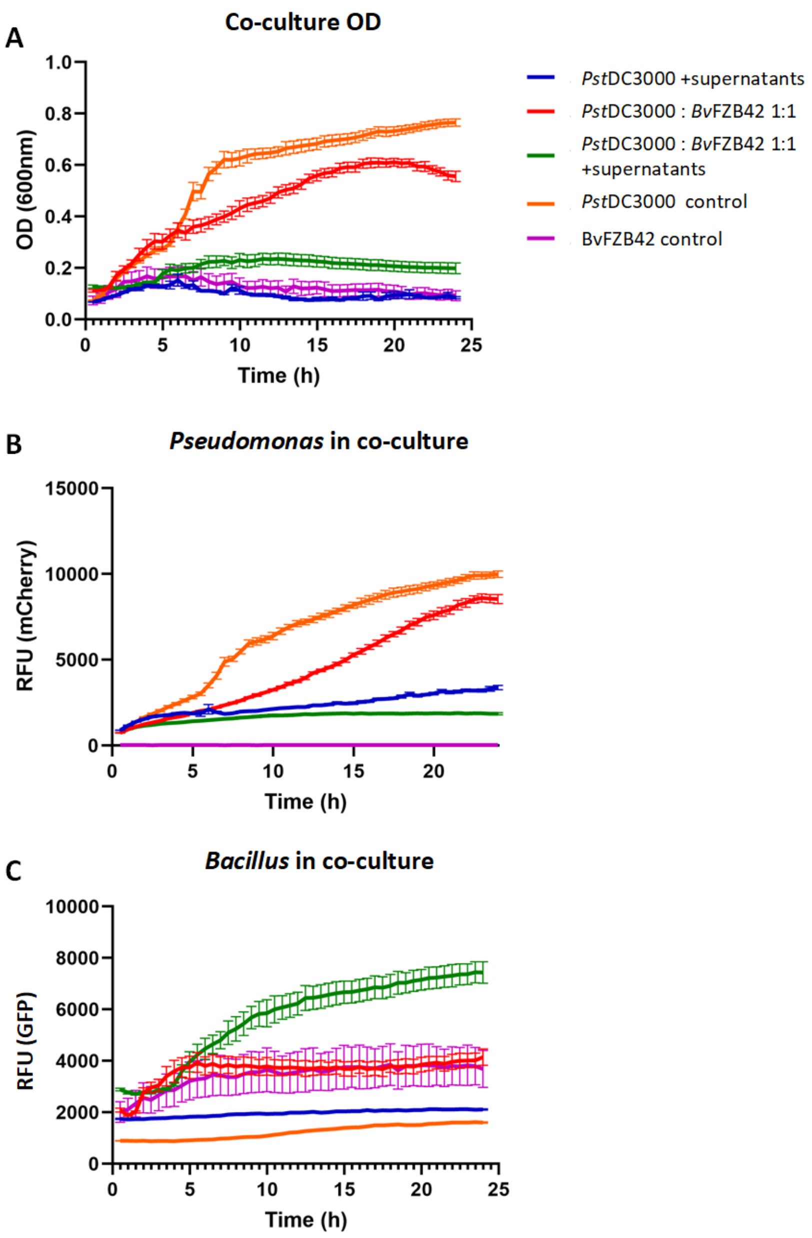

Fig. S12. Co-culture experiment of *Pst*DC3000 and *Bv*FZB42 under continuous shaking.

A, B and C present the growth curves of the five different treatments in liquid bulk conditions. Four repeats of 200 µL of each treatment were grown in a constantly shaking environment at 28°C in the plate reader (Synergy H1 Microplate Reader, BioTek Instruments, USA). Measurements were taken every 30 minutes for a total of 24 hours. OD (both bacteria) (A), mCherry (Growth of *Pst*DC3000 in co-culture) (B), and GFP (growth of *Bv*FZB42 in co-culture) (C) measurements were recorded. Lines and error bars represent mean ± SE.

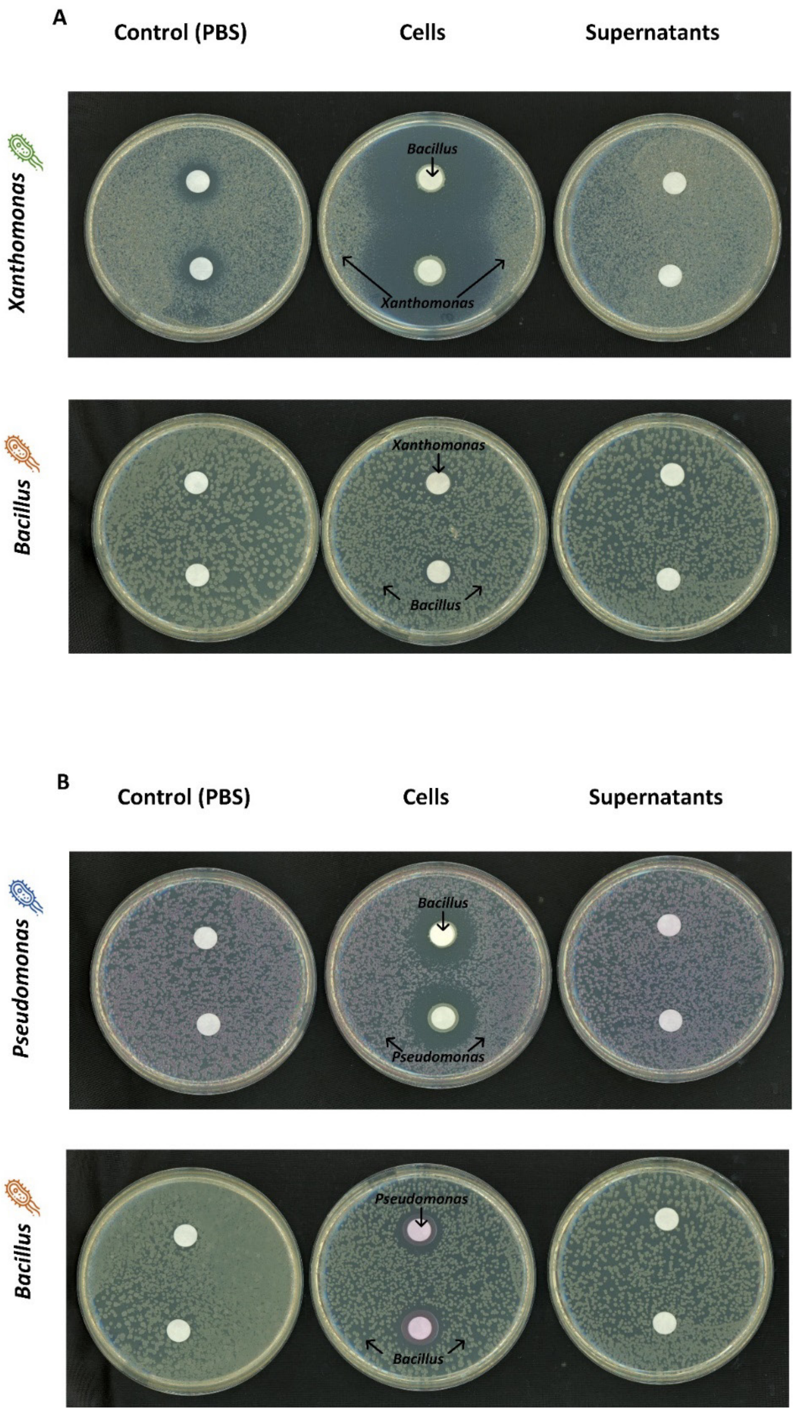

Fig. S13. Inhibition zone assay of *Xee*85-10 by *Bv*FZB42 and vice versa (A; top, bottom, respectively), and *Pst*DC3000 by *Bv*FZB42 and vice-versa (B; top, bottom, respectively).

*Xee*85-10 (OD_{600} =0.5), *Pst*DC3000m (OD_{600} =0.5) or *Bv*FZB42 (OD_{600} =0.5) were diluted by10^{-4} and 1 ml of the cell suspension was evenly spread onto LB-agar plates (130 mm). After a 1-hour incubation, discs containing either deionized water (diH_2O), supernatants or live bacterial cells (OD_{600}= 0.5) were carefully placed on the inoculated plates. Subsequently, the plates were incubated at 28°C for 3 days and the effectiveness of inhibition was qualitatively assessed by the presence of a clear area around the discs.

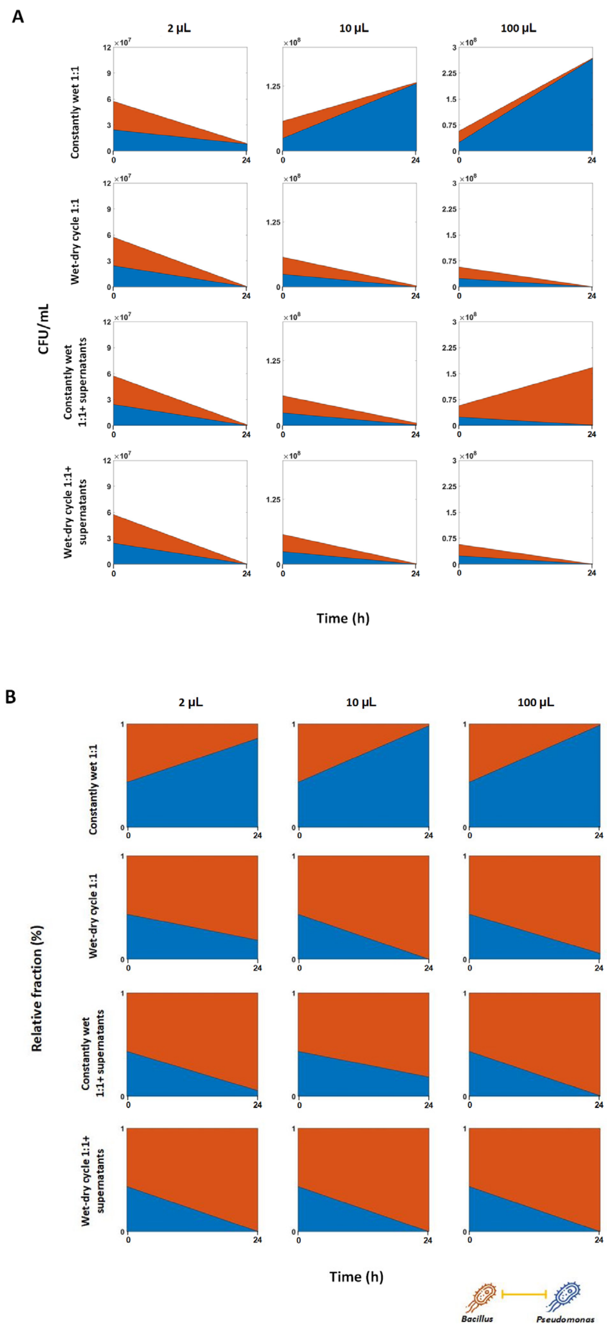

Fig. S14. Competition dynamics of *Pst*DC3000 and *Bv*FZB42 co-culture in droplets.

(A) stacked area plots and (B) relative part area plots in droplet co-cultures (1:1 and 1:1+ supernatants). The blue fraction represents *Pst*DC3000, and the orange fraction represents *Bv*FZB42. Note the plot is based on only two time points (at t=0 h and t=24 h).

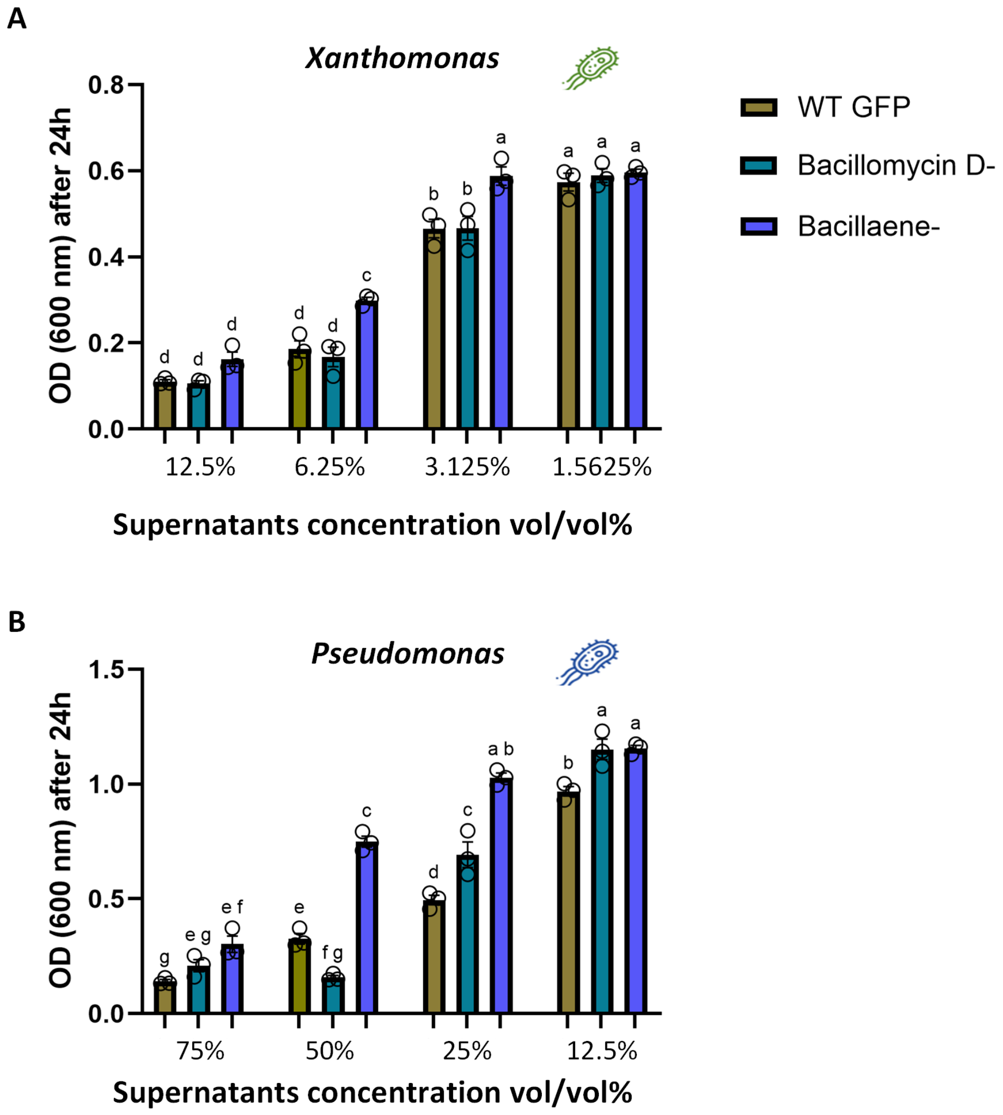

Fig. S15. Minimal Inhibitory Concentration assay of two antibiotic deficient mutants of *Bv*FZB42 on *Xee*85-10 (A) and *Pst*DC3000 (B).

OD_{600} was measured after 24 h following the addition of supernatants from GFP-tagged *Bv*FZB42 (WT GFP), Bacillomycin-D or Bacillaene deficient mutant strains of *Bv*FZB42. *Xee*85-10 at initial OD_{600}= 0.06 and *Pst*DC3000 at initial OD_{600}=0.2 were inoculated into a 96-well plate with supernatants at various dilutions (vol/vol%). Plates were kept at 220 rpm, at 28 °C for 24 hours and then OD was measured using a plate reader (Synergy H1 Microplate Reader, BioTek Instruments, USA). Bars and error bars represent mean ± SE OD_{600} after 24 hours. Black circles represent technical replicates The significance of the results was assessed using one-way ANOVA, followed by Tukey's post-hoc test for multiple comparisons. Statistically significant differences between groups are indicated by different letters, with groups sharing the same letter not being significantly different from one another ($p < 0.05$).

A

		polymyxin B		trimethoprim		chloramphenicol		neomycin		erythromycin		norfloxacin		carbenicillin	
		Ant Conc (μg/ml)	OD 24h (600 nm)	Ant Conc (μg/ml)	OD 24h (600 nm)	Ant Conc (μg/ml)	OD 24h (600 nm)	Ant Conc (μg/ml)	OD 24h (600 nm)	Ant Conc (μg/ml)	OD 24h (600 nm)	Ant Conc (μg/ml)	OD 24h (600 nm)	Ant Conc (μg/ml)	OD 24h (600 nm)
*Xee*8510	Rep1	20	0.112	10	0.523	20	0.17	10	0.601	20	0.182	2	0.318	100	0.306
		5	0.11	2.5	0.63	5	0.165	2.5	0.661	5	0.17	0.5	0.407	25	0.285
		1.25	0.11	0.625	0.625	1.25	0.174	0.625	0.558	1.25	0.234	0.125	0.561	6.25	0.376
		0.3125	0.595	0.15625	0.559	0.3125	0.539	0.15625	0.575	0.3125	0.482	0.03125	0.649	1.5625	0.411
	Rep 2	20	0.115	10	0.498	20	0.168	10	0.662	20	0.161	2	0.301	100	0.322
		5	0.114	2.5	0.55	5	0.176	2.5	0.677	5	0.199	0.5	0.292	25	0.387
		1.25	0.119	0.625	0.661	1.25	0.204	0.625	0.586	1.25	0.188	0.125	0.616	6.25	0.41
		0.3125	0.484	0.15625	0.616	0.3125	0.323	0.15625	0.532	0.3125	0.278	0.03125	0.599	1.5625	0.493
	Control	0	0.624		0.59		0.582		0.548		0.563				

B

		polymyxin B		trimethoprim		chloramphenicol		neomycin		erythromycin		norfloxacin		carbenicillin	
		Ant Conc (μg/ml)	OD 24h (600 nm)	Ant Conc (μg/ml)	OD 24h (600 nm)	Ant Conc (μg/ml)	OD 24h (600 nm)	Ant Conc (μg/ml)	OD 24h (600 nm)	Ant Conc (μg/ml)	OD 24h (600 nm)	Ant Conc (μg/ml)	OD 24h (600 nm)	Ant Conc (μg/ml)	OD 24h (600 nm)
*Pst*DC3000	Rep1	20	0.108	10	0.554	20	0.185	10	0.153	20	0.158	2	0.17	100	0.13
		5	0.132	2.5	1.034	5	0.226	2.5	0.272	5	0.199	0.5	0.269	25	0.527
		1.25	0.188	0.625	1.116	1.25	0.293	0.625	1.032	1.25	0.417	0.125	1.044	6.25	1.127
		0.3125	1.066	0.15625	1.099	0.3125	1.086	0.15625	1.113	0.3125	0.945	0.03125	1.125	1.5625	1.08
	Rep 2	20	0.126	10	0.599	20	0.18	10	0.205	20	0.186	2	0.174	100	0.166
		5	0.155	2.5	0.861	5	0.206	2.5	0.284	5	0.191	0.5	0.225	25	0.674
		1.25	0.215	0.625	1.138	1.25	0.242	0.625	1.003	1.25	0.275	0.125	0.867	6.25	0.99
		0.3125	1.04	0.15625	1.034	0.3125	0.864	0.15625	1.003	0.3125	0.815	0.03125	1.045	1.5625	1.006
	Control	0	1.228		1.216		1.3								

Fig. S16. Minimal inhibitory concentration (MIC) assay for *Xee*85-10 (A) and *Pst*DC3000 (B) using various antibiotics at fourfold serial dilutions.

*Xee*85-10 and *Pst*DC3000, starting at an initial OD_{600} of 0.06 and 0.2, respectively, were exposed to seven commercial antibiotics. The chosen antibiotics had various modes of action, most of which act similarly to antibiotics produced by *Bv*FZB42 (Table, S1). The colormap represents OD_{600} after 24 hours. Darker colors indicate higher OD values.

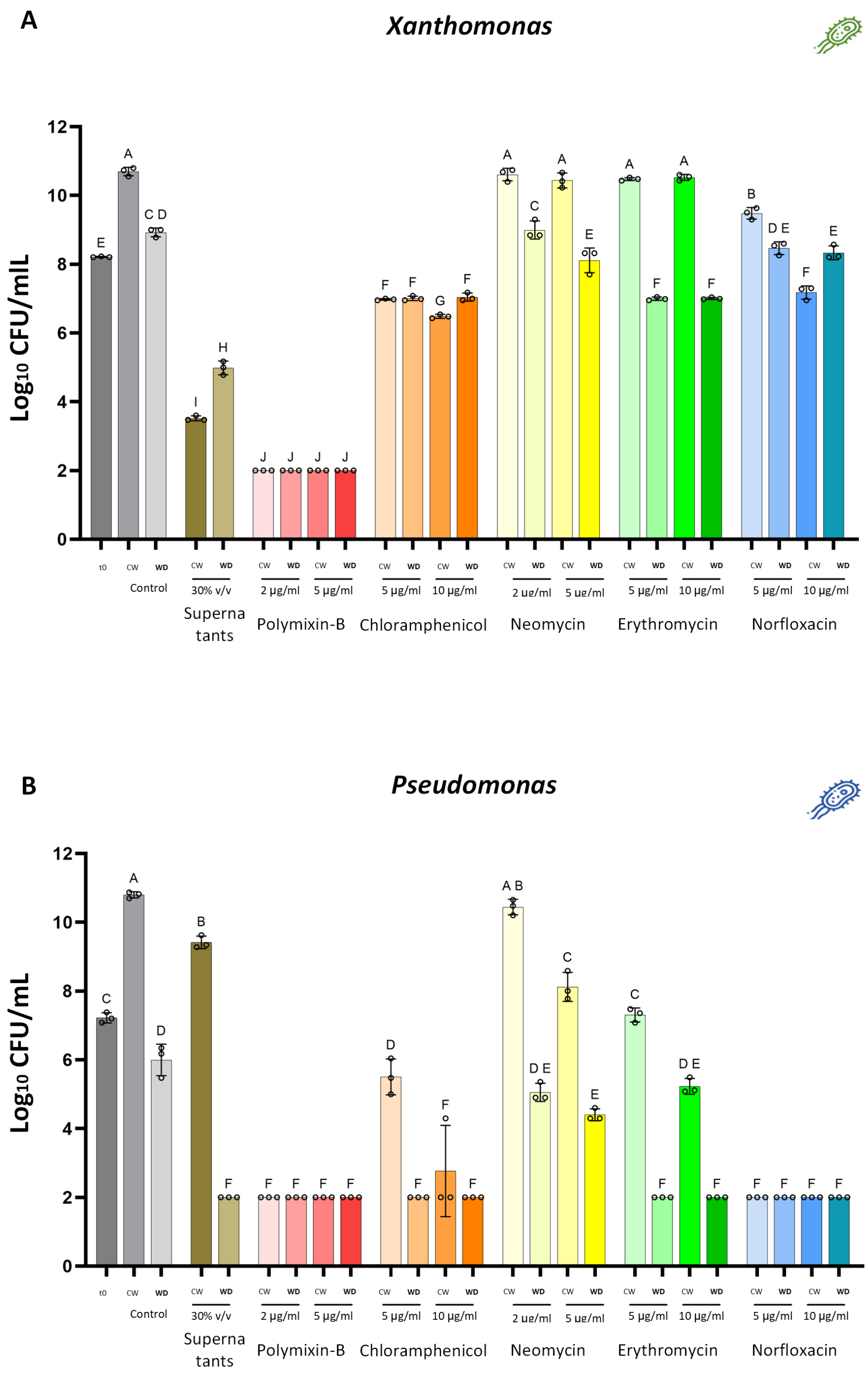

Fig. S17. ***Xee*85-10 (A) and *Pst*DC3000 (B) response to commercial antibiotics under constantly wet and wet-dry cycle conditions.**

Log $_{10}$ CFU/mL of *Xee*85-10 (A) and *Pst*DC3000 (B) exposed to different commercial antibiotics under constantly wet and wet-dry cycle conditions, at t=24 h. Assay was performed in 10 µL droplets. Antibiotic types and concentrations were chosen based on the observations presented in Fig. S16 and additional biological repeats of this MIC assay. Bars and error bars represent mean ± SE $\log_{10}$ CFU/mL. Black circles represent technical replicates. CFU measurements were conducted as described in Methods. The significance of the results was assessed using one-way ANOVA, followed by Tukey's post-hoc test for multiple comparisons. Statistically significant differences between groups are indicated by different letters, with groups sharing the same letter not being significantly different from one another ($p < 0.05$).

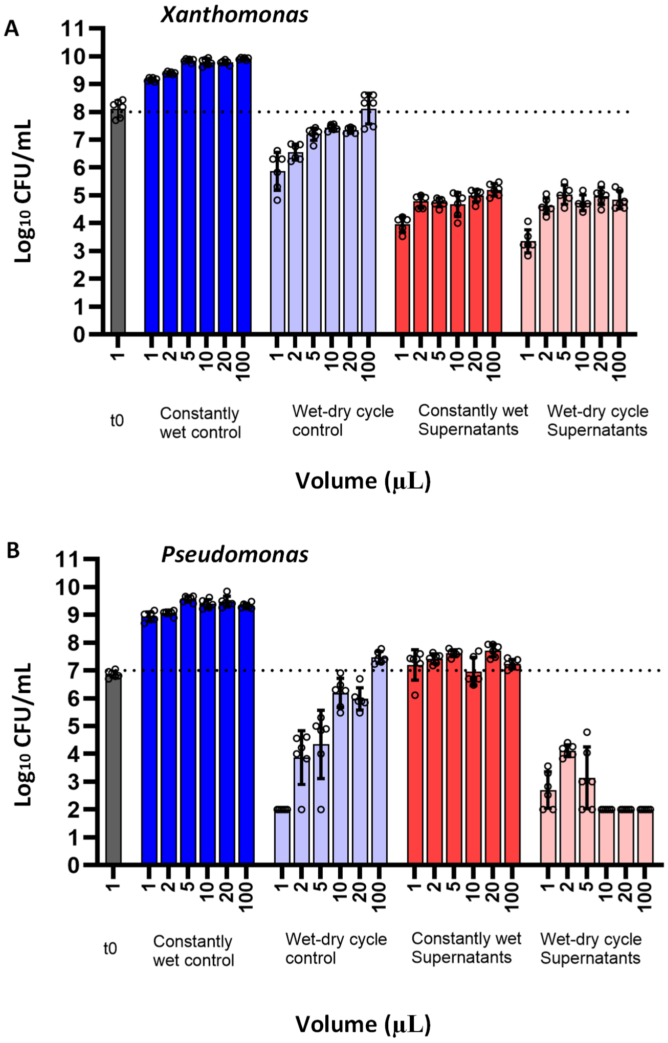

Fig. S18. ***Xee*85-10 (A) and *Pst*DC3000 (B) response to *Bv*FZB42 supernatants under wet-dry cycle and constantly wet conditions (Repetition of the entire experiment)**

Log_{10} CFU/mL of *Xee*85-10 (A) and *Pst*DC3000 (B) exposed to *Bv*FZB42 supernatants under constantly wet or wet-dry cycle conditions, at five different droplet volumes and at t=24 h,. The most left grey bar represents log_{10} CFU/mL at t=0 h. Bars and error bars represent mean ± SE log_{10} CFU/mL. Black circles represent technical replicates. CFU measurements were conducted as described in Methods.

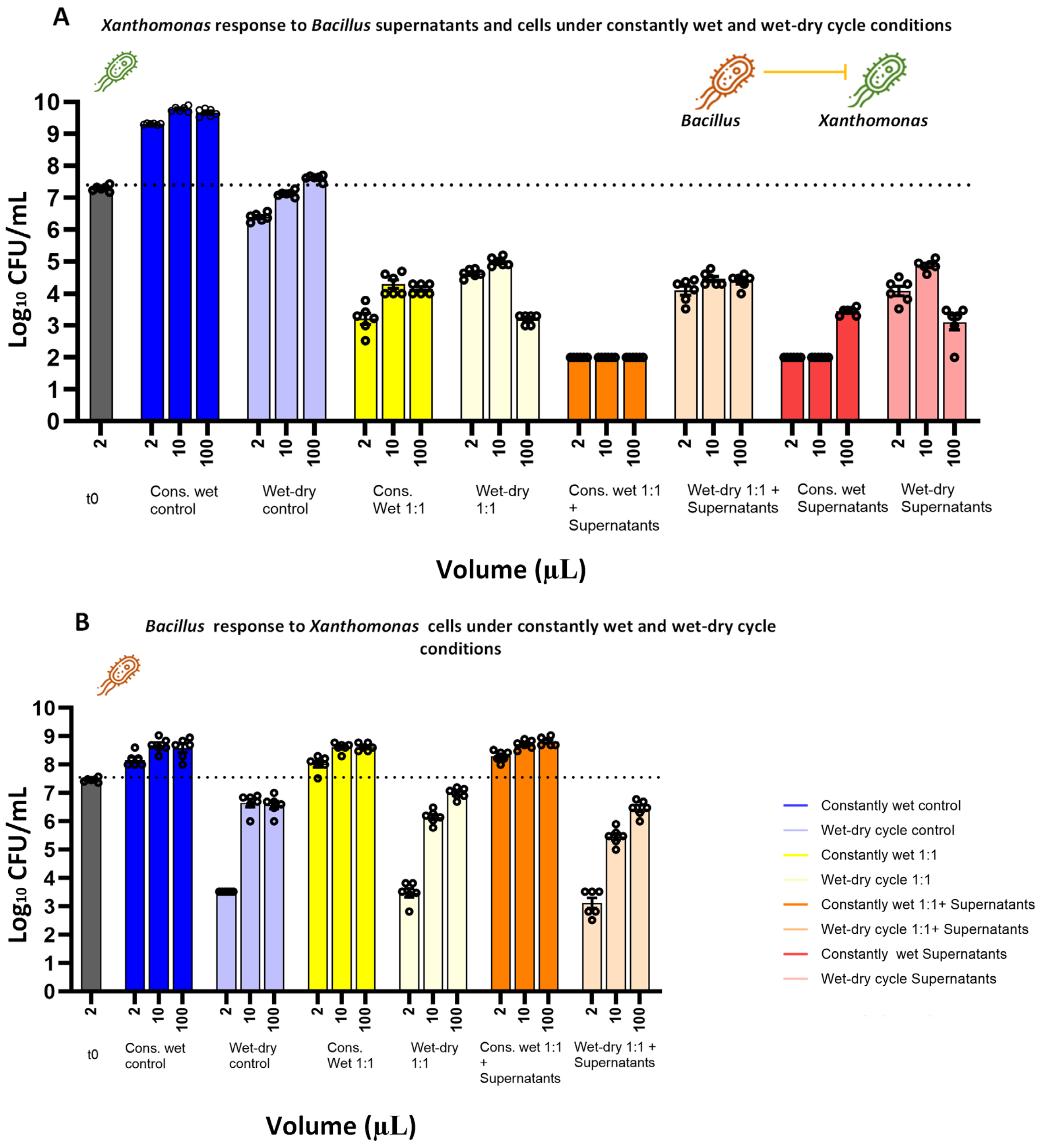

Fig. S19. Co-culture experiments of *Xee*85-10 and *Bv*FZB42 under constantly wet and wet-dry cycle conditions (Repetition of the entire experiment).

(A) Log_{10} CFU/mL of *Xee*85-10 at t=24 h under both constantly wet and wet-dry cycle conditions across different co-culture scenarios (equal ratio of both bacteria 1:1; equal ratio of both bacteria with *Bv*FZB42 supernatants 1:1+supernatants) and controls (*Xee*85-10 monoculture, *Bv*FZB42 monoculture, and *Xee*85-10 with *Bv*FZB42 supernatant) at three droplet volumes. The left grey bar represents log_{10} CFU/mL at t=0 h. Bars and error bars represent mean ± SE CFU/mL. Black circles represent technical replicates. **(B)** Same as in (A) but for *Bv*FZB42 (CFU/mL at t=24 h).

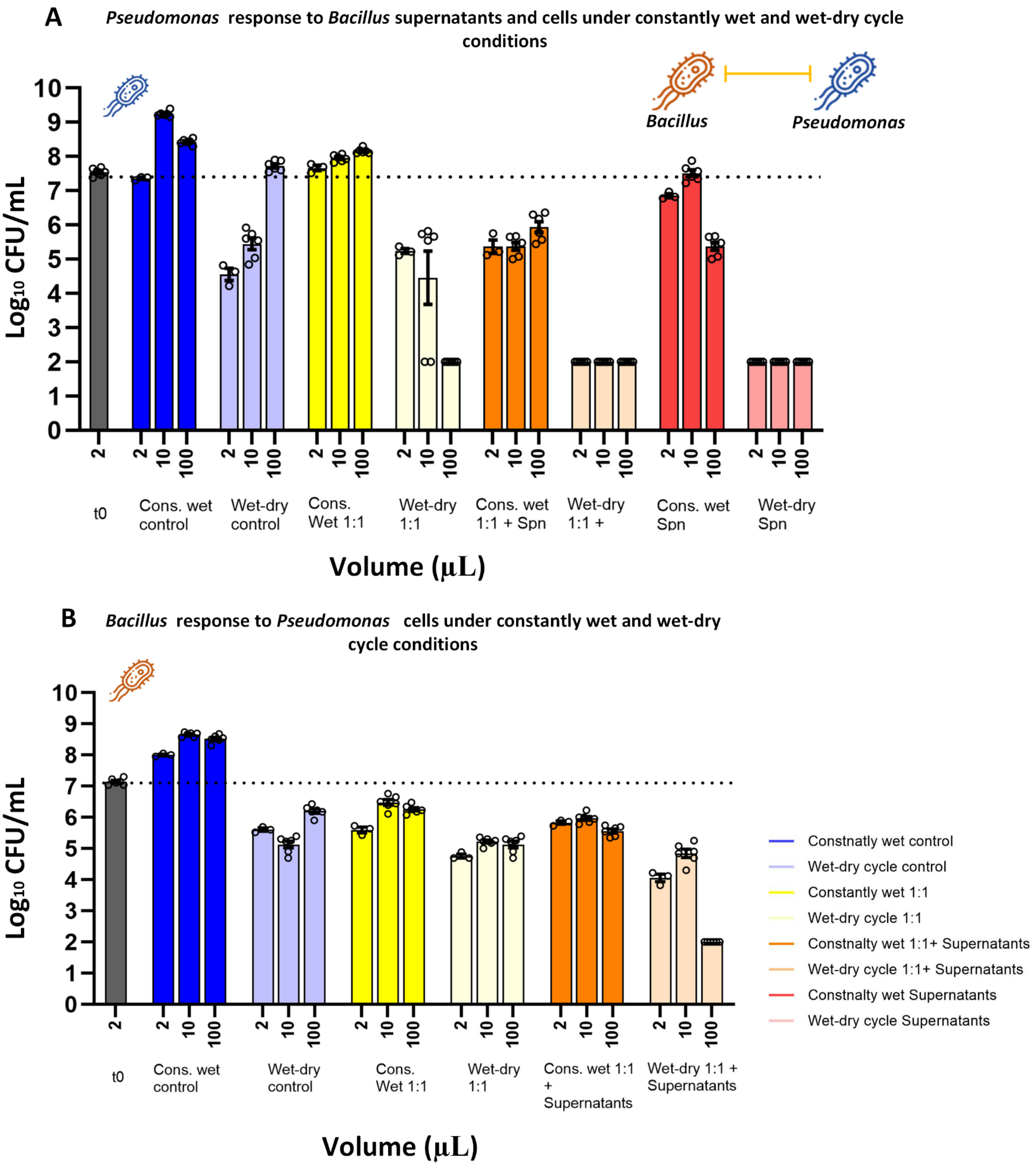

Fig. S20. Co-culture experiments of *Pst*DC3000 and *Bv*FZB42 under constantly wet and wet-dry cycle conditions (Repetition of the entire experiment).

(A) Log_{10} CFU/mL of *Pst*DC3000 at t=24 h under both constantly wet and wet-dry cycle conditions across different co-culture scenarios (equal ratio of both bacteria 1:1; equal ratio of both bacteria with *Bv*FZB42 supernatants 1:1+supernatants) and controls (*Pst*DC3000 monoculture, *Bv*FZB42 monoculture, and *Pst*DC3000 with *Bv*FZB42 supernatant) at three different droplet volumes. The left grey bar represents log_{10} CFU/mL at t=0 h. Bars and error bars represent mean ± SE CFU/mL. Black circles represent technical replicates. **(B)** Same as in (A) but for *Bv*FZB42 (CFU/mL at t=24 h).

References

1. Sheppard, J. D., Jumarie, C., Cooper, D. G. & Laprade, R. Ionic channels induced by surfactin in planar lipid bilayer membranes. *Biochim Biophys Acta* **1064**, 13–23 (1991).

2. Chen, X. *et al.* A mini-review: mechanism of antimicrobial action and application of surfactin. *World J Microbiol Biotechnol* **38**, 1–10 (2022).

3. Miller, W. R., Bayer, A. S. & Arias, C. A. Mechanism of Action and Resistance to Daptomycin in Staphylococcus aureus and Enterococci. *Cold Spring Harb Perspect Med* **6**, (2016).

4. Li, Z. & Velkov, T. Polymyxins: Mode of Action. *Adv Exp Med Biol* **1145**, 37–54 (2019).

5. Lv, J. *et al.* Mechanism of Antibacterial Activity of Bacillus amyloliquefaciens C-1 Lipopeptide toward Anaerobic Clostridium difficile. *Biomed Res Int* **2020**, 3104613 (2020).

6. Wu, T. *et al.* Bacillomycin D effectively controls growth of Malassezia globosa by disrupting the cell membrane. *Appl Microbiol Biotechnol* **104**, 3529–3540 (2020).

7. Zhang, L., Sun, C. & Master, E. R. Fengycins, Cyclic Lipopeptides from Marine Bacillus subtilis Strains, Kill the Plant-Pathogenic Fungus Magnaporthe grisea by Inducing Reactive Oxygen Species Production and Chromatin Condensation. *Appl Environ Microbiol* **84**, 445–463 (2018).

8. Wang, Y. *et al.* Iturin A Extracted From Bacillus subtilis WL-2 Affects Phytophthora infestans via Cell Structure Disruption, Oxidative Stress, and Energy Supply Dysfunction. *Front Microbiol* **11**, 536083 (2020).

9. Dertz, E. A., Xu, J., Stintzi, A. & Raymond, K. N. Bacillibactin-Mediated Iron Transport in Bacillus subtilis. *J Am Chem Soc* **128**, 22–23 (2006).

10. Page, M. G. P. Clinical Infectious Diseases The Role of Iron and Siderophores in Infection, and the Development of Siderophore Antibiotics. *Clinical Infectious Diseases ®* **69**, 529–566 (2019).

11. Islam, T., Rabbee, M. F., Choi, J. & Baek, K. H. Biosynthesis, Molecular Regulation, and Application of Bacilysin Produced by Bacillus Species. *Metabolites* **12**, 397 (2022).

12. Falagas, M. E., Vouloumanou, E. K., Samonis, G. & Vardakas, K. Z. Fosfomycin. *Clin Microbiol Rev* **29**, 321–347 (2016).

13. Pandey, N. & Cascella, P. Beta-Lactam Antibiotics. *StatPearls* (2019).

14. Yoo, J. S., Zheng, C. J., Lee, S., Kwak, J. H. & Kim, W. G. Macrolactin N, a new peptide deformylase inhibitor produced by Bacillus subtilis. *Bioorg Med Chem Lett* **16**, 4889–4892 (2006).

15. Siibak, T. *et al.* Erythromycin-and Chloramphenicol-Induced Ribosomal Assembly Defects Are Secondary Effects of Protein Synthesis Inhibition. *Antimicrob Agents Chemother* **53**, 563–571 (2009).

16. Mccoy, L. S., Xie, Y. & Tor, Y. Antibiotics that target protein synthesis. *Wiley Interdiscip Rev RNA* **2**, 209–232 (2011).

17. Tao, K. *et al.* In vivo and in vitro antibacterial activity of neomycin against plant pathogenic bacteria. *Scientific Research and Essays* **6**, 6829–6834 (2011).

18. Li, H. *et al.* Bacillaenes: Decomposition Trigger Point and Biofilm Enhancement in Bacillus. **6**, 1093–1098 (2021).

19. Fazle Rabbee, M. & Baek, K. H. Antimicrobial Activities of Lipopeptides and Polyketides of Bacillus velezensis for Agricultural Applications. *Molecules 2020, Vol. 25, Page 4973* **25**, 4973 (2020).

20. Carlos Molina-Santiago, A. *et al.* Chemical interplay and complementary adaptative strategies toggle bacterial antagonism and co-existence. *Cell Rep* **36**, (2021).

21. Bush, N. G., Diez-Santos, I., Abbott, L. R. & Maxwell, A. Quinolones: Mechanism, Lethality and Their Contributions to Antibiotic Resistance. *Molecules* **25**, 5662 (2020).

22. Wu, L. *et al.* Difficidin and bacilysin from Bacillus amyloliquefaciens FZB42 have antibacterial activity against Xanthomonas oryzae rice pathogens. *Scientific Reports 2015 5:1* **5**, 1–9 (2015).

23. Tran, C., Cock, I. E., Chen, X. & Feng, Y. Antimicrobial Bacillus: Metabolites and Their Mode of Action. *Antibiotics* **11**, 88 (2022).
